# A method for the allocation of sequencing resources in genotyped livestock populations

**DOI:** 10.1186/s12711-017-0322-5

**Published:** 2017-05-18

**Authors:** Serap Gonen, Roger Ros-Freixedes, Mara Battagin, Gregor Gorjanc, John M. Hickey

**Affiliations:** The Roslin Institute and Royal (Dick) School of Veterinary Studies, The University of Edinburgh, Easter Bush, Midlothian, Scotland, UK

## Abstract

**Background:**

This paper describes a method, called AlphaSeqOpt, for the allocation of sequencing resources in livestock populations with existing phased genomic data to maximise the ability to phase and impute sequenced haplotypes into the whole population.

**Methods:**

We present two algorithms. The first selects focal individuals that collectively represent the maximum possible portion of the haplotype diversity in the population. The second allocates a fixed sequencing budget among the families of focal individuals to enable phasing of their haplotypes at the sequence level. We tested the performance of the two algorithms in simulated pedigrees. For each pedigree, we evaluated the proportion of population haplotypes that are carried by the focal individuals and compared our results to a variant of the widely-used key ancestors approach and to two haplotype-based approaches. We calculated the expected phasing accuracy of the haplotypes of a focal individual at the sequence level given the proportion of the fixed sequencing budget allocated to its family.

**Results:**

AlphaSeqOpt maximises the ability to capture and phase the most frequent haplotypes in a population in three ways. First, it selects focal individuals that collectively represent a larger portion of the population haplotype diversity than existing methods. Second, it selects focal individuals from across the pedigree whose haplotypes can be easily phased using family-based phasing and imputation algorithms, thus maximises the ability to impute sequence into the rest of the population. Third, it allocates more of the fixed sequencing budget to focal individuals whose haplotypes are more frequent in the population than to focal individuals whose haplotypes are less frequent. Unlike existing methods, we additionally present an algorithm to allocate part of the sequencing budget to the families (i.e. immediate ancestors) of focal individuals to ensure that their haplotypes can be phased at the sequence level, which is essential for enabling and maximising subsequent sequence imputation.

**Conclusions:**

We present a new method for the allocation of a fixed sequencing budget to focal individuals and their families such that the final sequenced haplotypes, when phased at the sequence level, represent the maximum possible portion of the haplotype diversity in the population that can be sequenced and phased at that budget.

**Electronic supplementary material:**

The online version of this article (doi:10.1186/s12711-017-0322-5) contains supplementary material, which is available to authorized users.

## Background

This paper describes a method for the allocation of a fixed sequencing budget in a livestock population with existing phased genomic data. In livestock populations, the collection of marker array genotypes is routine but the collection of sequence data is less frequent. Collecting sequence data in a population can have a number of advantages. In human genetic studies, sequencing a large number of individuals was shown to increase the discovery of trait-associated and/or causative genetic variants using genome-wide association studies (GWAS) (e.g. [1–3]). In livestock, sequence data has enabled the discovery of causative mutations for qualitative traits (e.g. for embryonic lethality in the 1000 Bulls Project [4]), with only a few studies reporting some benefit for quantitative traits [5, 6]. To capture the full potential of sequence data in livestock, sequence and phenotype data on a large number, perhaps millions, of individuals may be required [7]. Only such large quantities of data will contain sufficient numbers of recombination events to accurately estimate the effects of large numbers of causative variants that underlie a quantitative trait.

The cost of generating large quantities of sequence data for millions of individuals is very high. Therefore, it is important to develop strategies that minimise the volume of data collected and maximise its utility for imputing sequence into the rest of the population [7–10]. One strategy could be to sequence a few key individuals and to impute their information into the rest of the population [7, 9]. For this strategy to be effective, a minimal group of key individuals whose genomes maximally represent the genetic diversity in the population must be selected and their haplotypes phased at the sequence level.

This raises two important questions: (1) how to select the key individuals to sequence, and (2) how to allocate a fixed sequencing budget among the key individuals to maximise the phasing accuracy of their haplotypes at the sequence level. Several solutions exist for the first of these problems but to our knowledge, no solution is available for the second or for the unification of the first and second problems.

Existing methods to select the key individuals to sequence fall into two broad categories: methods that use pedigree information and methods that use genomic information. Methods that use pedigree information identify the individuals with the largest pedigree-inferred marginal contributions in the population (i.e. the key ancestors approach) [4, 11–13]. Methods that use genomic information select individuals using heuristics [14] or by inferring shared haplotypes using existing genomic data [15, 16]. The key sires approach is sub-optimal in that it does not explicitly account for the realised sharing of haplotypes across a population and does not account for the information required for accurate phasing of haplotypes at the marker array and sequence level, which is essential for subsequent imputation. Algorithms that select individuals based on realised haplotype sharing such as those presented in Bickhart et al. [15] and Gusev et al. [16] can account for these factors but do so in slightly different ways. The algorithm of Bickhart et al. [15] attempts to select the least redundant set of individuals that represent the population haplotypes by scoring haplotypes based on their frequency, whereas the algorithm of Gusev et al. [16] attempts to select individuals that share a large proportion of the population haplotypes with other individuals identical-by-descent (IBD).

The common aim of all existing methods is to capture the maximum amount of population diversity in only a subset of the population. To our knowledge, all methods assume that selected individuals would be sequenced at a set coverage and do not consider the ability to phase the sequenced haplotypes. When the aim of sequencing a subset of the population is to enable sequence imputation into non-sequenced members of the population, sequencing all selected individuals at a set coverage is likely to be sub-optimal since it does not guarantee phasing of haplotypes at the sequence level, which is essential for subsequent sequence imputation. In structured livestock populations, accurate phasing could be achieved using simple inheritance-based rules if some of the sequencing resource is allocated to the parents and grandparents of selected individuals, which is something that current optimisation algorithms do not do.

This creates a need to develop methods that jointly address both the selection of key individuals to sequence and the allocation of a fixed sequencing budget with the aim of maximising the proportion of population haplotypes sequenced and phased at the sequence level. A method for selecting the key individuals could capitalise on existing genotype, haplotype or sequence data to infer realised relationships between individuals in the population. When allocating the fixed sequencing budget, the method must: (1) account for the frequency of all haplotypes of a key individual in the population; (2) account for the impact that phasing these haplotypes would have for sequence imputation into the rest of the population; and (3) maximise the ability to phase haplotypes at the sequence level.

In this paper, we propose a new and fast method for the allocation of a fixed sequencing budget across a population that implements two algorithms. The first algorithm selects ‘focal individuals’ to sequence. A focal individual is one that shares a large number of its own haplotypes with a large number of individuals in the population, and need not be a key ancestor and may have no offspring. The second algorithm allocates a fixed sequencing budget across focal families (i.e. a focal individual, its two parents and four grandparents). A fixed sequencing budget is referred to in monetary terms. We tested the performance of the two algorithms in simulated pedigrees and compared it to the key ancestors approach as implemented in the PEDIG software [17] and to two haplotype-based approaches, that of Bickhart et al. [15] and Gusev et al. [16]. We show that compared to the other methods, our method selects focal individuals whose haplotypes are more frequent in the population. By allocating part of the sequencing budget to the relatives of focal individuals, we show that a large proportion of the sequenced haplotypes could be phased at the sequence level.

## Methods

### Description of the method

We present two algorithms. The first selects ‘focal individuals’ whose genomes collectively represent the maximum possible portion of the haplotype diversity in the population. A focal individual is one that shares a large number of its own haplotypes with a large number of individuals in the population. This individual does not need to be a key ancestor, and may have no offspring. The second allocates a fixed sequencing budget across focal families (i.e. a focal individual, its parents and grandparents) to enable phasing of the most frequent haplotypes in the population at the sequence level. The aim of both algorithms is that the final haplotypes, when phased at the sequence level, represent the maximum possible portion of the haplotype diversity in the population that can be sequenced and phased for a fixed sequencing budget. We implemented our method in a software package called AlphaSeqOpt. Throughout the rest of the paper, AlphaSeqOpt is used when referring to our method. An outline of each algorithm is given below.

#### Algorithm 1: select and rank the focal individuals

 Input data: existing phased, true or imputed genotype, haplotype or sequence data.

1. For each chromosome, determine a set of $m$ haplotypes of length $n$ markers.
2. Determine the haplotypes of each individual and construct a population haplotype library. Calculate the frequency of each haplotype in the population.
3. Select a focal individual that carries more of the most frequent haplotypes.
4. Mask the haplotypes of this focal individual in the rest of the population, assuming that sequencing and phasing its haplotypes would enable sequence imputation of non-sequenced individuals that share these haplotypes.
5. Repeat steps 2 to 4 to generate a list of the $k$ focal individuals for sequencing. Since $k$ is user-defined, this process can be repeated until all haplotypes in the population would be sequenced.

#### Algorithm 2: allocation of a fixed sequencing budget across focal families

To help phase and resolve the haplotypes of focal individuals, some of the fixed sequencing budget should be allocated to an additional group of individuals that share their haplotypes. In populations with pedigree, the best additional group of individuals is likely to be the parents and grandparents of focal individuals. The advantage of sequencing parents and grandparents is that simple inheritance-based rules can be developed to phase the sequenced haplotypes of focal individuals. To maximise the accuracy of phasing the most frequent haplotypes in a population, a larger proportion of the fixed sequencing budget could be allocated to the families of focal individuals whose haplotypes are more frequent in the population, and this is addressed by algorithm 2.

The inputs for algorithm 2 are:

- The $k$ focal individuals and the proportion of population haplotypes that each one carries.
- The accuracies of phasing each member of a focal family given a chosen ‘sequencing scenario’, i.e. the selected sequencing coverage for each member of a focal family. Expected phasing accuracies for a sequencing scenario may be calculated using algorithms such as that implemented in AlphaFamSeq (Battagin and Hickey, unpublished), which has been developed by our group specifically for family-based phasing of haplotypes at the sequence level (for a brief description, see Additional file 1: Figure S1).
- Population pedigree.
- The cost of preparing and sequencing a DNA library at any coverage.
- The total fixed sequencing budget (in monetary terms).
- Information on any historically available sequence data.

The allocation of a fixed sequencing budget in algorithm 2 is addressed using a differential evolution algorithm [18] that samples and evaluates different combinations of sequencing scenarios within and across the focal families. Algorithm 2 can be run for any number of rounds until convergence is reached or until no further improvements are made. An outline of algorithm 2 is given below.

- For each member of a focal family, sample a sequencing coverage and determine the sequencing scenario and haplotype phasing accuracy. Sampling of sequencing coverages is performed based on the multinomial probabilities of sequencing an individual at a defined coverage. The probabilities are obtained by logit transforms of the internal problem representation in the differential evolution algorithm [18]. Haplotype phasing accuracies given a sequencing scenario were calculated using AlphaFamSeq (Battagin and Hickey, unpublished) (see Additional file 1: Figure S1 for more information).
- Calculate the overall cost of the selected set of sequencing scenarios across all focal families, taking into account pre-existing DNA libraries and/or sequence data.
- Compute a ‘goodness criterion’ for this combination of sequencing scenarios and associated cost. The criterion takes into account:
- The proportion of sequenced population haplotypes that would be phased at the sequence level.
- The accuracy of phasing the haplotypes of focal individual given sampled sequencing scenarios [AlphaFamSeq (Battagin and Hickey, unpublished)].
- The fixed sequencing budget. If the total cost is above the budget, then this combination of sequencing scenarios is penalised.
- Any historically available sequence data.
- Repeat steps 1 to 3 $n$ times ($n$ is the number of rounds).

The final result is an ordered list of focal individuals that carry the highest proportion of the most frequent haplotypes in the population and the sequencing coverage for each focal individual, its parents and grandparents. For further details on both algorithms, see Additional file 2.

### Examples of method implementation: description of datasets

To demonstrate the implementation of the algorithms, testing datasets were simulated to obtain genotype and pedigree information for six different populations. The first five resembled livestock populations with known structured pedigrees, and the sixth resembled a population of unrelated individuals, which could represent a natural population or some livestock populations. A generalised description of each population is given below and summarised in Table 1.

**Table 1:** Summary of the parameters used to simulate the six populations

Populations	Number of generations	Number of individuals
1	5	6000
2	10	11,000
3	15	16,000
4	30	31,000
5	50	51,000
6	Unrelated	100,000

#### Genotypic data

Sequence data was generated for 1000 base haplotypes for each of ten chromosomes using the Markovian Coalescent Simulator [19] and AlphaSim [20, 21]. Chromosomes were simulated as 100 centiMorgans (cM) and 10^8^ bp in length, with a per site mutation rate of 2.5 × 10^−8^ and a per site recombination rate of 1.0 × 10^−8^. The effective population size ($N_{e}$) was set at specific points during the simulation based on previously estimated $N_{e}$ values within the Holstein cattle population [22]. These set points were: 100 in the base generation, 1256 at 1000 years ago, 4350 at 10,000 years ago, and 43,500 at 100,000 years ago, with linear changes in between. The resulting sequence had approximately 650,000 segregating sites across the ten chromosomes.

### Quantitative trait nucleotides

To enable the selection of parents to generate a pedigree in populations 1 to 5, a quantitative trait influenced by 10,000 quantitative trait nucleotides (QTN) that are distributed equally across the ten chromosomes was simulated. QTN positions were randomly chosen from the 650,000 segregating sites and their effect sizes sampled from a normal distribution with a mean of zero and variance of 1.0 divided by the number of QTN. QTN effects were used to compute the true breeding value (TBV) for each individual.

### Generation of a pedigree

To emulate the recent history of modern livestock breeding, ten replicates of five pedigrees were simulated. Pedigrees were 5, 10, 15, 30 or 50 generations for populations 1 to 5, respectively. All pedigrees and replicates were independently simulated and had the following general structure. Each generation comprised 1000 individuals with equal sex ratio, i.e. 500 males and 500 females. In the first generation, chromosomes for each individual were sampled from the 1000 haplotypes in the base generation. In subsequent generations, chromosomes of each individual were sampled from parental chromosomes, assuming recombination with no interference. In each generation, the 25 males with the highest TBV were selected as sires of the next generation. No selection was performed on females, and all 500 females were used as parents. The sixth population was simulated to obtain an unrelated population of 100,000 individuals directly from base haplotypes, i.e. individuals were nominally unrelated. This population was simulated to test the performance of the algorithm in extreme circumstances. Circumstances such as these may not typically arise in livestock breeding but could arise in human or other natural populations or in gene bank collections, which are especially topical in plant breeding (e.g. the Seeds of Discovery project, CIMMYT: http://seedsofdiscovery.org/). We assumed that all individuals had genotypes for 10,000 single nucleotide polymorphisms (SNPs) distributed equally across the ten chromosomes, i.e. 1000 SNPs per chromosome. Genotypes of all individuals were phased using AlphaPhase [23–25] as input. We also performed the same analysis with a pedigree from a real livestock breeding program. Since the results showed the same trends as the simulated data and in the interests of brevity, these results are not presented.

### Selection and ranking of focal individuals

In each population, the parameters for selecting the focal individuals were:

- Population haplotype libraries were created using individuals and SNPs with at least 90% phased genotype data.
- Sharing of haplotypes was determined as 100% identity matches. A 100% identity match was chosen to overcome phasing errors and ensures that haplotypes with small differences are considered as independent haplotypes.
- Haplotype lengths were set to 250 SNPs per chromosome (see Additional file 3 for haplotype length choice).

For each population, we calculated the frequency of all haplotypes of the top 50 and 200 focal individuals selected by algorithm 1 in the population. For populations 1 to 5, we compared this to the frequency of all haplotypes of the top 50 and 200 focal individuals selected by the key ancestors approach and two haplotype-based approaches. We implemented the key ancestors approach using PEDIG [17], which selects focal individuals that cumulatively have the largest pedigree-inferred marginal contributions. The two haplotype-based approaches were those by Bickhart et al. [15] and Gusev et al. [16]. The algorithm of Bickhart et al. [15] attempts to select the least redundant set of individuals that represent the population haplotypes by scoring haplotypes based on their population frequency, whereas the algorithm of Gusev et al. [16] attempts to select individuals that share a large proportion of the population haplotypes with other individuals IBD.

### Allocate the fixed sequencing budget

For populations 1 to 5 with pedigree, we calculated the proportion of the fixed sequencing budget to allocate to each of the top 50 focal families using algorithm 2. The possible sequencing coverages were 0, 1, 2, 5, 10 and 20x. Sequencing each member of a focal family at one of the above coverages gave a possible number of 281,728 sequencing scenarios, thus the algorithm had to find the best combination of sequencing scenarios across the 50 focal families out of a possible (281,728)^50^ combinations. The algorithm was run for 10,000 rounds.

We calculated the cost of each sequencing scenario assuming a DNA library cost of £40 GBP and 1x sequencing cost of £85 GBP. The expected haplotype phasing accuracy given a sequencing scenario was calculated using simulated genotype and sequence data and AlphaFamSeq (Battagin and Hickey, unpublished); (for a brief overview of AlphaFamSeq, see the description for Additional file 1: Figure S1). The expected haplotype phasing accuracy for a focal individual at the sequence level against the cost of a sequencing scenario is plotted in Additional file 1: Figure S1. Additional file 1: Figure S1 shows that increasing the sequencing budget allocated to a focal family increases the phasing accuracy, but the same phasing accuracy may also be achieved with a lower cost sequencing scenario. Therefore, there is a benefit in choosing the sequencing scenario for each focal family given a fixed sequencing budget for the population. Using this information, we calculated the combination of sequencing scenarios for the top 50 focal families at four budgets of £50,000 GBP, £75,000 GBP, £100,000 GBP and £150,000 GBP.

## Results

The results show that Algorithm 1 in AlphaSeqOpt selects focal individuals that carry more of the haplotypes in the population than the key ancestors approach and the two haplotype-based methods by Bickhart et al. [15] and Gusev et al. [16]. Since the three existing methods do not account for the ability to phase sequenced haplotypes, we were unable to compare the performance of Algorithm 2 of AlphaSeqOpt with them. The results from Algorithm 2 show that the unequal distribution of a fixed sequencing budget across focal individuals and their families could enable phasing of a large proportion of the population haplotypes at the sequence level.

For ease of presentation, we have split the results into two sections to demonstrate the implementation and benefits of each algorithm individually. We present algorithm 1 in light of sequencing the top 50 and 200 focal individuals and algorithm 2 in light of sequencing the top 50 focal individuals only. In the description of the results, we use the terms ‘closely related’ to refer to individuals that share recent common ancestors and ‘distantly related’ to refer to individuals that do not share recent common ancestors (i.e. >10 generations apart). We use the terms ‘shallow’ or ‘deep’ to refer to the size of the pedigree. We refer to the Bickhart et al. [15] algorithm as ‘Bickhart’ and to the Gusev et al. [16] algorithm as ‘Gusev’.

### Algorithm 1: selection and ranking of focal individuals

#### Maximizing the proportion of population haplotypes sequenced

**Fig. 1:**
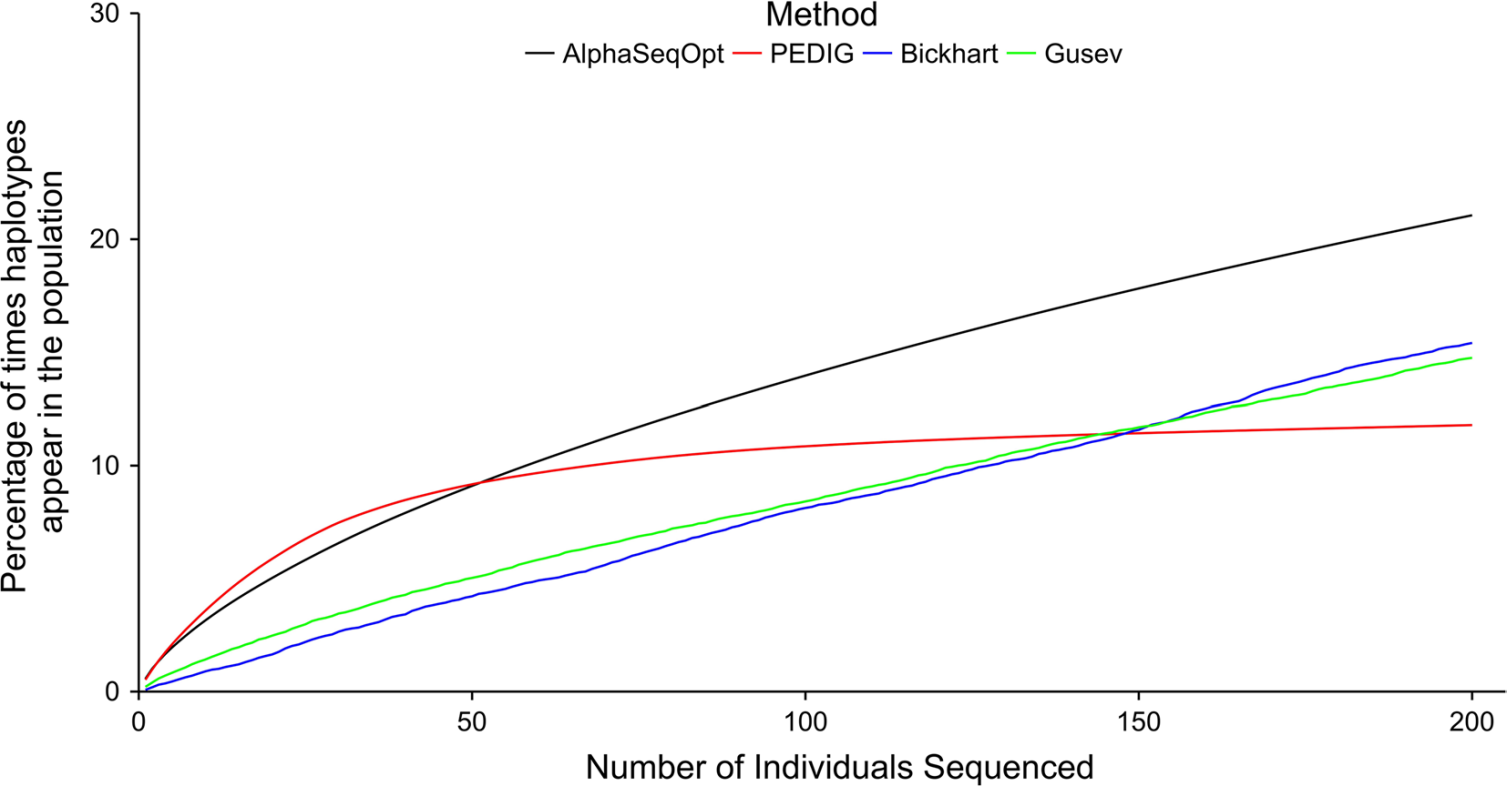
Comparison of cumulative percentage of haplotypes carried by individuals selected by the four methods. A comparison of the cumulative percentage of haplotypes carried by the top 200 focal individuals selected by AlphaSeqOpt, the key ancestors approach, Bickhart et al. [15] or Gusev et al. [16] against the number of individuals sequenced for the 30-generation pedigree

AlphaSeqOpt selects focal individuals whose haplotypes are more frequent in the population than existing methods. This is shown in Fig. 1, which plots the cumulative frequency of the haplotypes of the top 200 focal individuals selected by AlphaSeqOpt, the key ancestors approach or the two haplotype-based methods of Bickhart and Gusev against the number of focal individuals sequenced for the 30-generation pedigree only. The values for all pedigrees for the top 50 and 200 focal individuals are in Additional file 4: Tables S1 and S2. These values are standardised according to the total number of individuals in each pedigree. The cumulative sum of the pedigree-inferred marginal contributions of the top 50 and 200 focal individuals selected by the key ancestors approach is in Additional file 4: Table S3.

Averaging across all pedigrees, the haplotypes of the top 50 focal individuals selected by AlphaSeqOpt are 1.44 times more frequent than those selected by the key ancestors approach (5.84 vs. 4.05% of the population), 1.20 times more frequent than those selected by Bickhart (5.84 vs. 4.87%), and 1.02 times more frequent than those selected by Gusev (5.84 vs. 5.68%). This frequency ratio, i.e. ratio of the frequencies in the population of the haplotypes carried by the selected individuals, is a convenient way to compare different selection methods and strategies. If we consider the top 200 focal individuals, the advantage increases furthermore: the frequency ratio is 1.90 (21.25 vs. 11.16%) compared to the key ancestor approach, 1.20 (21.25 vs. 17.89%) compared to Bickhart and 1.22 (21.25 vs. 17.40%) compared to Gusev.

#### Distribution of the top 50 and 200 focal individuals in the pedigree

**Fig. 2:**
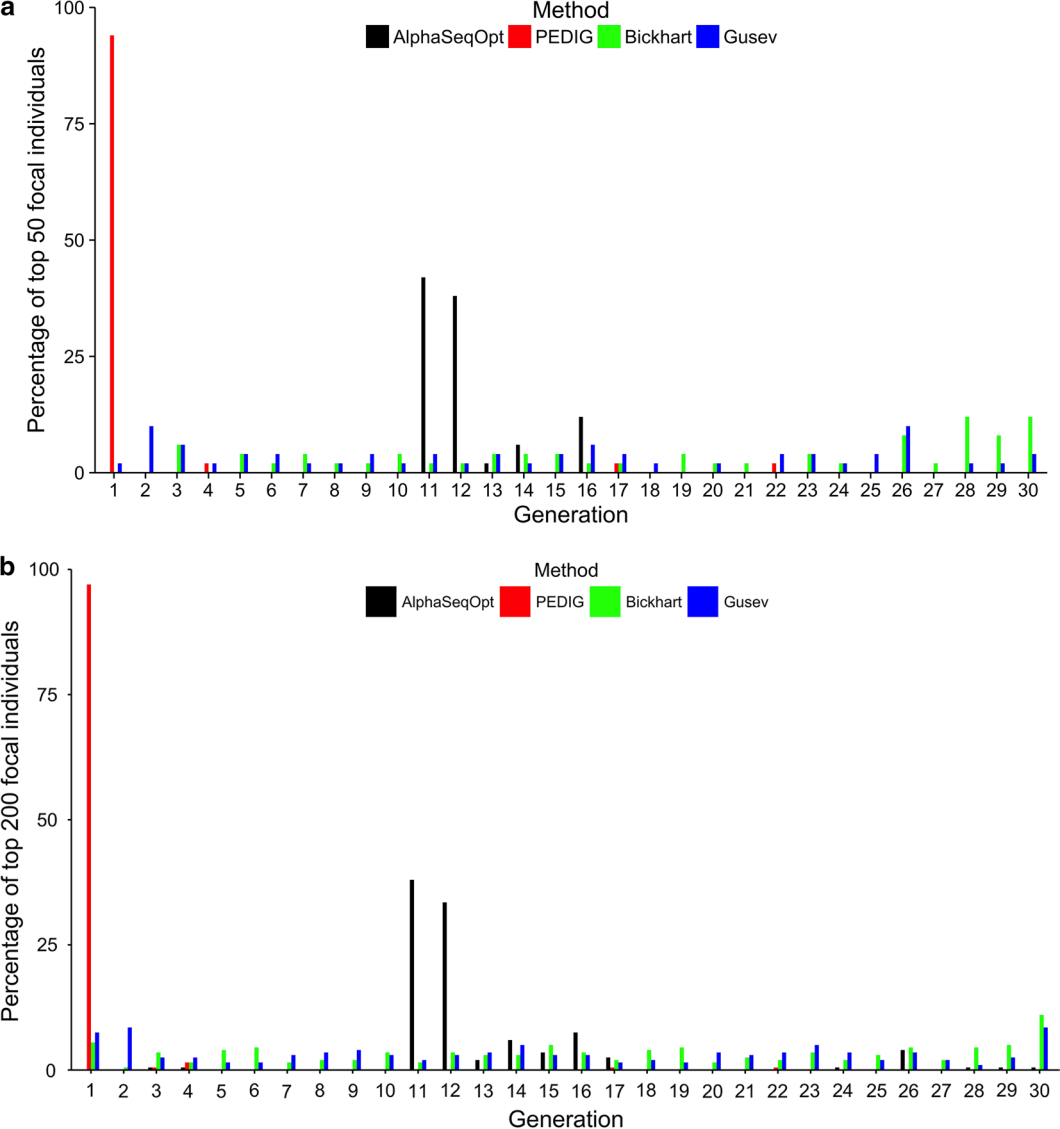
Comparison of the positions of selected focal individuals in the pedigree. Percentage of the **a** top 50 and **b** top 200 focal individuals selected by AlphaSeqOpt, the key ancestors approach, Bickhart et al. [15] or Gusev et al. [16] against the number of generations for the 30-generation pedigree

AlphaSeqOpt selects focal individuals mainly from the middle generations of the pedigree whereas the key ancestors approach selects mainly from the oldest generations of the pedigree. The two haplotype-based methods of Bickhart and Gusev select focal individuals from across the pedigree, but with more emphasis on the older and younger generations. This is shown in Fig. 2, which plots the percentage of the top (a) 50 and (b) 200 focal individuals against generation for the mid-range 30-generation pedigree (the same diagrams for all other pedigrees are Additional file 5: Figures S2, S3, S4, S5 and S6 for the top 200 focal individual only). We can see this if we consider the generation from which the majority of the top focal individuals originate for each method. Figure 2 shows that approximately 50% of the top 50 focal individuals selected by AlphaSeqOpt were from generation 11 and 94% selected by the key ancestors approach were from generation 1. For the two haplotype-based methods by Bickhart and Gusev, almost all generations had at least one individual selected, but approximately 50% were either from the first seven or last five generations. Approximately 50% of the top 200 focal individuals selected by AlphaSeqOpt were from generations 11 and 12 and 97% selected by the key ancestors approach were from generation 1. As above for the two haplotype-based methods by Bickhart and Gusev, almost all generations had at least one individual selected, but the majority were from the first six or last seven generations.

#### Effect of relatedness on maximizing the proportion of haplotypes sequenced

**Fig. 3:**
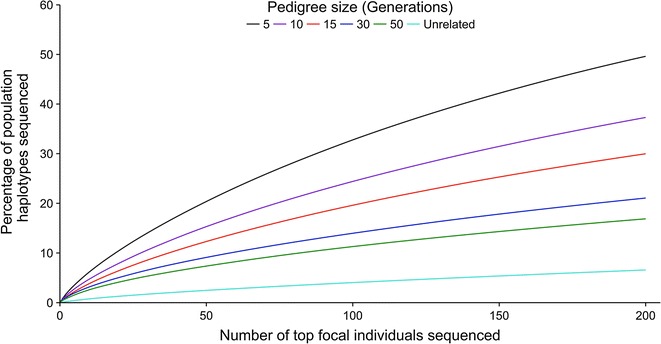
Percentage of population haplotypes that would be sequenced in the individuals selected by AlphaSeqOpt. Percentage of population haplotypes that would be sequenced by sequencing the top 200 focal individuals selected by AlphaSeqOpt against the number of focal individuals sequenced for pedigrees of 5, 10, 15, 30 and 50 generations and for the unrelated population

In populations with some relatedness, AlphaSeqOpt selects focal individuals that carry a higher proportion of the population haplotypes than in unrelated populations, but the difference decreases with increasing pedigree depth. This is shown in Fig. 3, which plots the cumulative proportion of population haplotypes that are carried by the top focal individuals against the number of focal individuals for pedigrees of 5, 10, 15, 30 and 50 generations and for the unrelated population. This is clear from the order of the curves in Fig. 3 and we can compare the curves more precisely by looking at the ratio of the proportion of haplotypes in the related compared to the unrelated population. If we consider the top 50 focal individuals, in the 5-generation pedigree the ratio is 8.24 (20.36 vs. 2.47%), in the 30-generation pedigree the ratio is 3.69 (9.11 vs. 2.47%) and in the 50-generation pedigree it is 2.98 (7.36 vs. 2.47%). These ratios only decrease slightly, if we consider the top 200 individuals, and were equal to 7.54 in the 5-generation pedigree (49.63 vs. 6.58%), 3.20 in the 30-generation pedigree (21.06 vs. 6.58%) and 2.56 in the 50-generation pedigree (16.87 vs. 6.58%).

Figure 3 also shows that in shallow pedigrees, focal individuals that carry a higher proportion of the population haplotypes are selected than in deep pedigrees. This is clear when we compare the ratio of the proportion of haplotypes in the 5-generation pedigree to the 30- and 50-generation pedigrees. If we consider the top 50 focal individuals, the ratio is equal to 2.24 when compared to the 30-generation pedigree (20.36 vs. 9.11%) and 2.77 when compared to the 50-generation pedigree (20.36 vs. 7.36%). These ratios are consistent when we consider the top 200 focal individuals and were equal to 2.36 when compared to the 30-generation pedigree (49.63 vs. 21.06%) and 2.94 when compared to the 50-generation pedigree (49.63 vs. 16.87%).

### Algorithm 2: allocation of the fixed sequencing budget

In populations with pedigree data, the sequenced haplotypes of a focal individual could be phased if some of the fixed sequencing budget is allocated to its parents and grandparents. The proportion of the budget to allocate to each focal family is addressed in algorithm 2. For ease of presentation, we have split the results into two parts: (1) the allocation of £100,000 GBP, and (2) the allocation of varying budgets.

#### Fixed sequencing budget of £100,000 GBP

##### Realised proportion of haplotypes phased at the sequence level

**Fig. 4:**
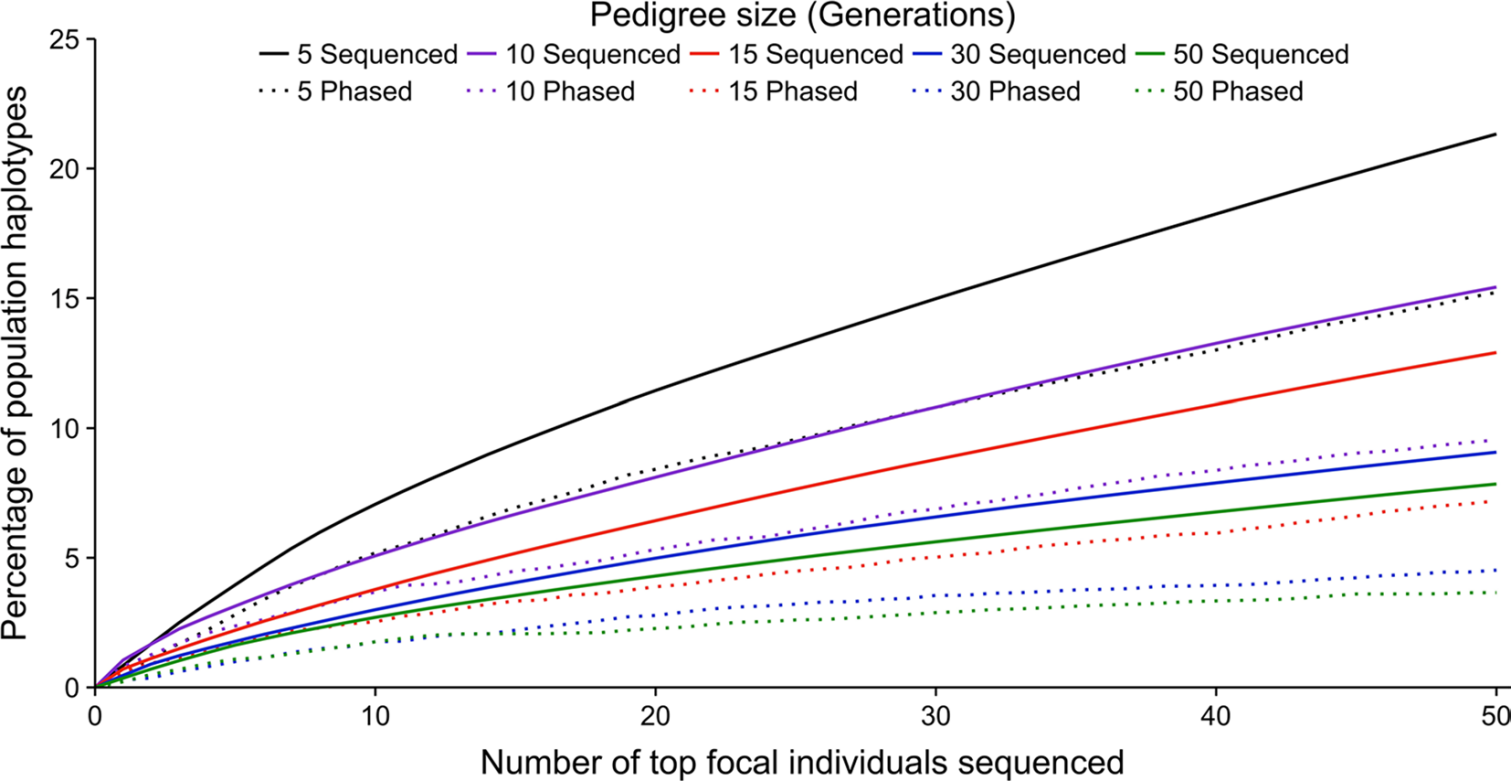
Percentage of population haplotypes that would be sequenced and phased with £100,000 GBP. Expected percentage of population haplotypes carried by the top 50 focal individuals that would be sequenced (*solid lines*) and phased at the sequence level at a fixed sequencing budget of £100,000 GBP (*dashed lines*) against the number of focal individuals sequenced for pedigrees of 5, 10, 15, 30 and 50 generations

AlphaSeqOpt calculates the proportion of the fixed sequencing budget to allocate to each focal family so as to maximise the phasing accuracy of sequenced haplotypes, but is more effective in shallow pedigrees. This is shown in Fig. 4, which plots the expected proportion of population haplotypes carried by the top 50 focal individuals that would be phased against the number of focal families sequenced for a fixed sequencing budget of £100,000 GBP. The expected maximum proportion of population haplotypes carried by the top 50 focal individuals are the solid lines and the expected proportion of these haplotypes that would be phased at the sequence level are the dashed lines. In the 5-generation pedigree, 71.36% of the sequenced population haplotypes carried by the top 50 focal individuals would be phased at the sequence level (21.33% of population haplotypes would be sequenced and 15.22% would be phased), 49.85% would be phased in the 30-generation pedigree (9.07 vs. 4.52%), and 46.57% would be phased in the 50-generation pedigree (7.85 vs. 3.65%).

##### Accounting for shared ancestry

**Fig. 5:**
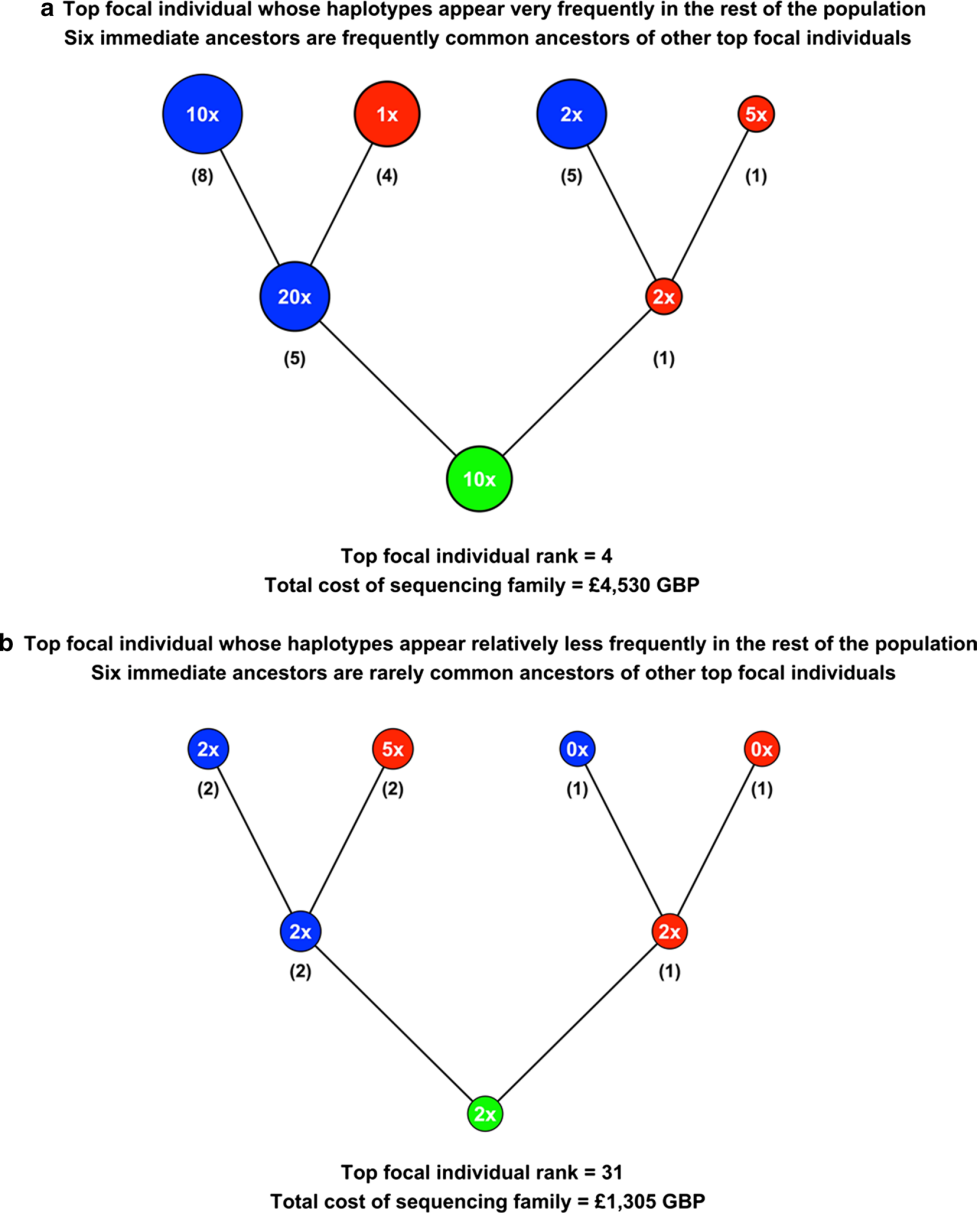
Comparison of budget allocation in two families that carry different proportions of the population haplotypes. **a** This panel shows a focal individual whose haplotypes are very frequent in the population and whose six immediate ancestors are frequently ancestors of other focal individuals. **b** This panel shows a focal individual whose haplotypes are relatively less frequent in the population and whose six immediate ancestors are rarely ancestors of other focal individuals. For each family member, the chosen sequencing coverage at a fixed sequencing budget of £100,000 GBP (text in the *circles* indicating each individual) and the number of times it is an ancestor of another focal individual (*numbers* in *brackets*) are given

More of the fixed sequencing budget is allocated to the families of focal individuals whose haplotypes are more frequent in the population than to those whose haplotypes are less frequent. An example of this is given in Fig. 5, which is a diagram showing (a) a focal individual whose haplotypes are very frequent and whose six immediate ancestors are frequently common ancestors of other focal individuals, and (b) a focal individual whose haplotypes are relatively less frequent and whose six immediate ancestors are rarely ancestors of other focal individuals. The magnitude of the unequal allocation of the fixed sequencing budget can be quantified by considering the ratio of the amount of money allocated to family (a) compared to family (b). Figure 5 shows that at the family level, this ratio is equal to 3.47 (total costs of £4530 GBP for 50x coverage vs. £1305 GBP for 13x coverage). For the focal individual itself, this ratio is equal to 4.24 (£890 GBP for 10x vs. £210 GBP for 2x). For the parents and grandparents, the ratios are equal to 4.64 and 2.50 (£1950 GBP for 22x vs. £420 GBP for 4x for the parents; £1690 GBP for 18x versus £675 GBP for 7x for the grandparents). This unequal allocation would enable more accurate phasing of the haplotypes of the focal individual whose haplotypes are more frequent and would improve the ability to impute sequence into the rest of the population.

##### Choice of ancestor to sequence at high coverage

**Fig. 6:**
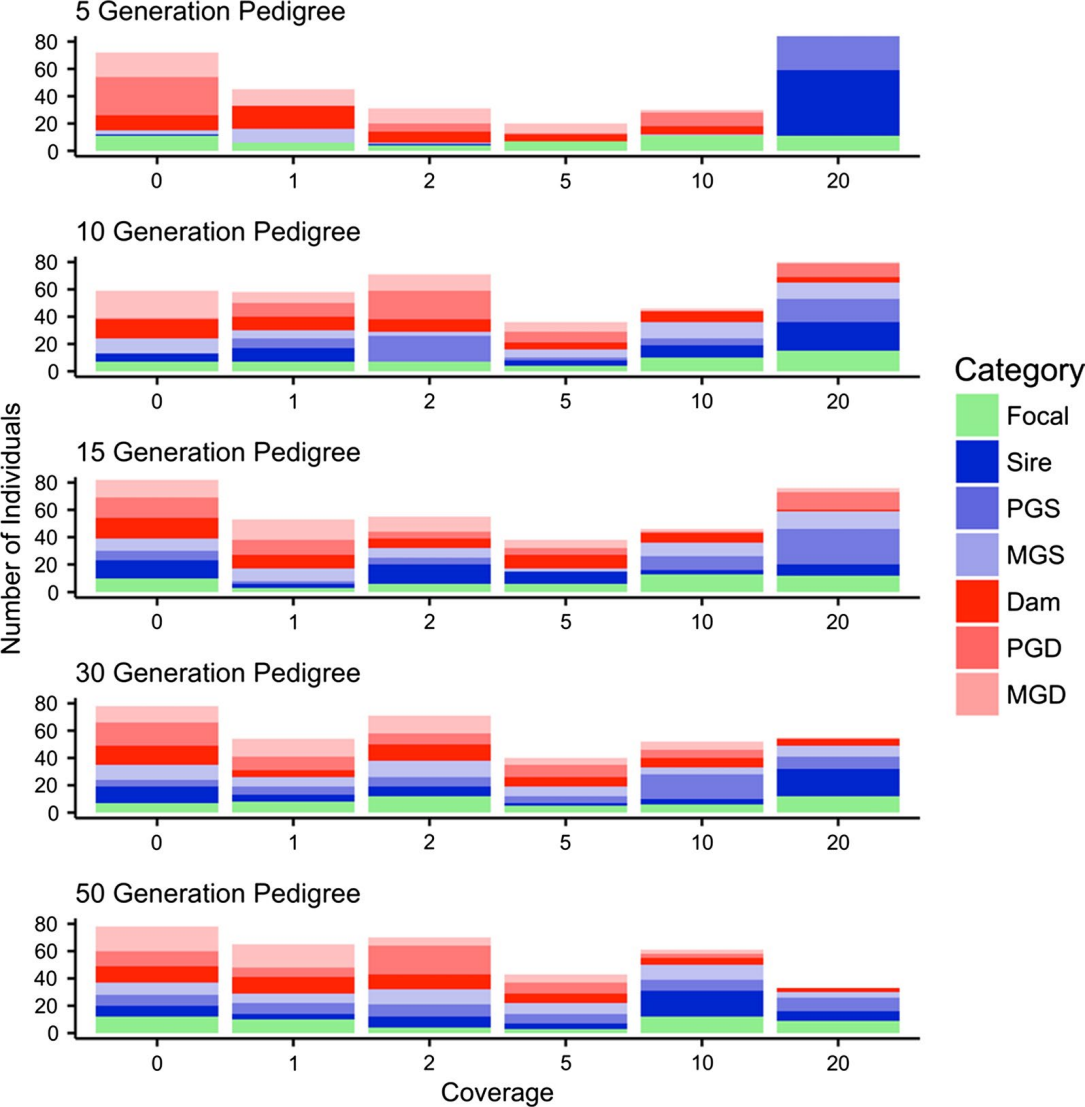
Distribution of sequencing coverage across focal families with £100,000 GBP. Number of focal individuals, sires, dams and paternal and maternal grandparents against sequencing coverage for the top 50 focal families selected from pedigrees of 5, 10, 15, 30 and 50 generations

The fixed sequencing budget is allocated so that some individuals are sequenced at high coverage while some individuals are not sequenced at all. This is shown in Fig. 6, which plots the number of focal individuals, sires, dams and grandparents against sequencing coverage in the top 50 focal families for pedigrees of 5, 10, 15, 30 and 50 generations. For example, 18% of individuals were sequenced at 20x (34 of 188 individuals) and 25% were not sequenced (47 of 188) in the 5-generation pedigree, 11% were sequenced at 20x (25 of 228) and 25% were not sequenced (56 of 228) in the 30-generation pedigree and 6% were sequenced at 20x (17 of 271) and 25% were not sequenced (69 of 271) in the 50-generation pedigree.

Figure 6 also shows that in shallow pedigrees, sires and grandsires were mainly selected to be sequenced at the highest coverage of 20x compared to deep pedigrees. We can see this by considering the proportion of individuals selected for sequencing at 20x that were sires or paternal/maternal grandsires. This proportion was 88% in the 5-generation pedigree, 67% in the 30-generation pedigree and 64% in the 50-generation pedigree. In addition, regardless of the pedigree depth, dams and granddams were generally selected for sequencing at 2x or less. We can see this by considering the proportion of individuals selected for sequencing at 2x or less that were dams or paternal/maternal granddams. This proportion was 75% in the 5-generation pedigree, 51% in the 30-generation pedigree and 54% in the 50-generation pedigree.

#### Varying sequencing budgets

##### Realised proportion of haplotypes phased at the sequence level

**Fig. 7:**
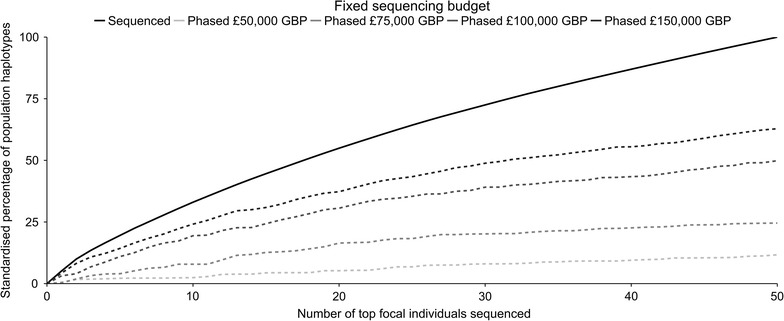
Percentage of population haplotypes that would be sequenced and phased with four different budgets. Expected percentage of population haplotypes that would be sequenced (*solid line*) and phased at the sequence level (*dashed lines*) against the number of focal individuals sequenced for fixed sequencing budgets of £50,000 GBP, £75,000 GBP, £100,000 GBP and £150,000 GBP. The figure shows the case for the top 50 focal individuals selected from the mid-range 30-generation pedigree. All values have been standardised by the proportion of population haplotypes that would be sequenced by sequencing the top 50 focal individuals (i.e. the *solid line* reaches 100%)

When the fixed sequencing budget is large, sequencing scenarios with better haplotype phasing accuracy are selected. This is shown in Fig. 7, which plots the proportion of the population haplotypes that are carried by the top 50 focal individuals selected from the mid-range 30-generation pedigree that would be phased at the sequence level against the number of focal families sequenced for budgets of £50,000 GBP, £75,000 GBP, £100,000 GBP and £150,000 GBP. This is clear from the order of the curves in Fig. 7 and we can compare the curves by looking at the ratio of the proportion of the haplotypes captured in the top 50 focal individuals that would be phased at the highest budget of £150,000 GBP compared to the other budgets. The ratio was equal to 1.26 when compared to £100,000 GBP (62.83 vs. 49.85%), 2.56 when compared to £75,000 GBP (62.83 vs. 24.55%) and 5.38 when compared to £50,000 GBP (62.83 vs. 11.68%). Figure 7 also shows that doubling the fixed sequencing budget more than doubles the percentage of population haplotypes that would be phased at the sequence level. The ratio was equal to 4.27 when the budget was doubled from £50,000 GBP to £100,000 GBP (11.68 vs. 49.85%), and 2.56 from £75,000 GBP to £150,000 GBP (24.55 vs. 62.83%).

##### Number of individuals sequenced

**Fig. 8:**
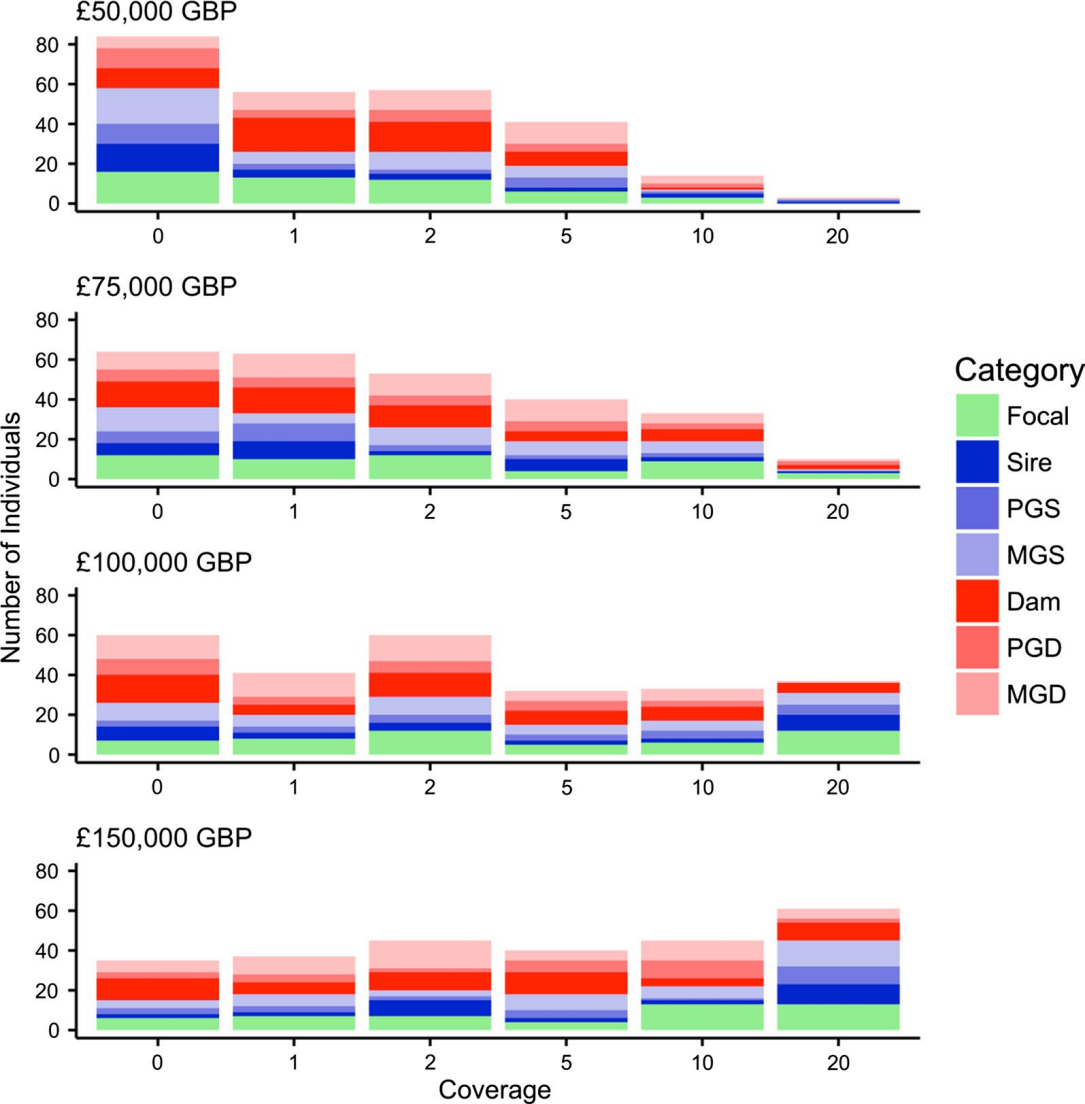
Distribution of sequencing coverage across focal families with varying budgets. Number of focal individuals, sires, dams and paternal and maternal grandparents against sequencing coverage for the top 50 focal families selected from the mid-range 30-generation pedigree. The figure shows four panels representing fixed sequencing budgets of £50,000 GBP, £75,000 GBP, £100,000 GBP and £150,000 GBP

When the fixed sequencing budget is large, slightly more individuals are selected for sequencing at a coverage of 1x or more and more individuals are selected for sequencing at 20x coverage than when the budget is small. This is shown in Fig. 8, which is similar to Fig. 6 and shows the number of focal individuals, sires, dams and grandparents against sequencing coverage for the top 50 focal families selected from the mid-range 30-generation pedigree. Figure 8 has four panels, one for each fixed sequencing budget of £50,000 GBP, £75,000 GBP, £100,000 GBP and £150,000 GBP. We can see this clearly when we consider the effect of doubling the sequencing budget on the ratio of the number of individuals sequenced. When the budget was doubled from £50,000 GBP to £100,000, the ratios were equal to 1.03 with at least 1x (173 vs. 178) and 12.33 at exactly 20x (3 vs. 37) and when it was doubled from £75,000 GBP to £150,000 GBP they were equal to 1.06 with at least 1x (180 vs. 191) and 6.10 at exactly 20x (10 vs. 61).

## Discussion

The results highlight four main points for discussion: (1) the advantages of AlphaSeqOpt over existing methods; (2) factors affecting performance; (3) downstream applications of sequence data; and (4) potential use cases.

### Advantages over existing methods

In this study, we compared the performance of Algorithm 1 in AlphaSeqOpt with three existing methods and showed that AlphaSeqOpt performed better in all cases tested. The first method used for the comparison was the key ancestors approach as implemented in PEDIG, which selects key ancestors that cumulatively have the largest marginal contributions inferred using pedigree relationships only. The second and third methods, that of Bickhart et al. [15] and Gusev et al. [16], are more similar to AlphaSeqOpt in that they use existing genomic data to select individuals based on realised sharing of haplotypes. Our results show that AlphaSeqOpt outperforms all three methods in the proportion of population haplotypes that could be sequenced. We discuss these advantages furthermore below.

If no genomic data is available and cannot be generated, then the key ancestors approach is a useful way of selecting individuals that may be most representative of the haplotype diversity in the population. However, when genomic data is available, methods that use them such as AlphaSeqOpt and the two haplotype-based methods of Bickhart et al. [15] and Gusev et al. [16] are expected to be more powerful and our results support this. In our simulated datasets, AlphaSeqOpt was able to capture slightly more (in general approximately 1.20 times more) of the population haplotypes in the same number of individuals compared to the algorithms of Bickhart et al. [15] and Gusev et al. [16]. This slight advantage of AlphaSeqOpt may be because it explicitly accounts for the realised Mendelian sampling of an individual, the haplotype inheritance across generations, haplotype sharing between individuals and explicitly uses common features of livestock populations such as known close relationships between families and the large genetic footprint of males on the population caused by the high selection intensity on males. Although the methods of Bickhart et al. [15] and Gusev et al. [16] do these things implicitly to some degree, it is perhaps the explicitness of AlphaSeqOpt that gives it its advantage.

Regardless of the way in which the subset of individuals is selected, the generated sequence data is of little value for sequence imputation into non-sequenced individuals if the constituent haplotypes cannot be phased at the sequence level. In our view, the fact that AlphaSeqOpt addresses this directly, is its major advantage over existing methods. To our knowledge, all of the existing methods that select individuals to be sequenced assume equal sequencing coverage across all individuals. In contrast, Algorithm 2 of AlphaSeqOpt distributes a fixed sequencing coverage across selected individuals and their immediate ancestors with the aim of maximising the ability to phase haplotypes carried by the sequenced individuals. The allocation of some of the fixed sequencing budget to immediate ancestors of focal individuals has an additional advantage in that sequence data will be available for more individuals, and so more of the population haplotypes will be sequenced and potentially phased to some extent.

In Algorithm 2, we assume that the ancestors of focal individuals are known and that all family members have DNA available. This means that Algorithm 1 is more likely to select individuals from later generations with more complete pedigree and genomic information. In comparison, the key ancestors approach (and to some extent, the haplotype-based methods by Bickhart et al. [15] and Gusev et al. [16]) select from older generations. This is important in livestock breeding programs that have been running for several decades with pedigrees that extend for many generations (e.g. a 20- to 30-generation pedigree in cattle or swine), where sequencing individuals from older generations is unlikely to be useful for sequence imputation in the younger generations. This is because (i) DNA samples for older individuals are often unavailable or the quality of DNA is not high enough for whole-genome sequencing, (ii) ancestors of older individuals are usually unknown and the individuals themselves are by design unrelated and do not share haplotypes amongst themselves (which makes phasing of their haplotypes more difficult), and (iii) the many generations of meiosis and recombinations separating older individuals and the imputation targets in younger generations reduce the expectation of finding shared long-range haplotypes. Selecting individuals from across the pedigree whose ancestors are more likely to be known will improve the ability to phase haplotypes and increase the likelihood of finding shared haplotypes with both older and younger generations, thus improving the ability to impute sequence into more of the population.

### Factors affecting performance

The proportion of haplotypes captured and phased depends on the structure of the population and dataset and is influenced by the availability of phased genomic data for all individuals, the degree of relatedness between individuals, and the number of rounds. We discuss these factors further below.

#### Availability of phased genomic data for all individuals

Algorithm 1 uses existing phased genomic (i.e. genotype, haplotype or sequence) data to infer sharing of haplotypes between individuals. This assumes that phased genomic data is available or can be accurately imputed for all individuals in the population, which is the case for many livestock populations (e.g. [4]). However, these datasets will generally exclude individuals from the older generations if tissue or DNA samples for these individuals do not exist or because these individuals are no longer important for informing breeding decisions in the younger generations. In this case, focal individuals from younger generations may be selected, which is advantageous since they are more likely to have good quality DNA or tissue samples available, are more likely to carry the most frequent haplotypes segregating in the current breeding population, and any haplotypes and sequence variants identified using their sequence data will be most relevant and are likely to be still segregating in the younger generations. This is particularly important if the sequence data will be used for sequence imputation and for informing breeding decisions in younger generations.

#### Degree of relatedness

In populations in which individuals are closely related, the majority of the population haplotypes may be captured by sequencing a few focal families in which individuals are also ancestors in other non-sequenced families. In this case, a larger proportion of the fixed sequencing budget could be allocated to these few focal families and the individuals that are shared ancestors of other families. This would enable more accurate phasing of the shared ancestor’s haplotypes at the sequence level, increase the accuracy of phasing the haplotypes of focal individuals that share this ancestor, and improve sequence imputation.

In shallow pedigrees, focal individuals whose haplotypes are more frequent in the population are selected compared to deep pedigrees. The magnitude of this difference may be an overestimate caused by the long haplotype length, which we set to a quarter of the chromosome across all pedigrees. A better solution may be to choose haplotype lengths according to the population structure and degree of relatedness between individuals. This would allow the selection of focal individuals so as to maximise the use and benefit of the sequence data for imputation in a target set of individuals. For example, short haplotype lengths may bias selection and be more beneficial for imputation in older generations, whereas long haplotype lengths may be more relevant for selection from and imputation in younger generations. If the individuals in a population are unrelated, short haplotype lengths may be favored.

#### Number of rounds

Internally, algorithm 2 is a differential evolution algorithm [18] that samples and evaluates different combinations of sequencing scenarios for the focal families. The number of rounds required for convergence of the algorithm depends on the number and possible combinations of sequencing scenarios that may be sampled. The number and possible combinations of sequencing scenarios depend on the number of focal families selected for sequencing and the number of possible sequencing scenarios.

Currently, focal families are considered for sequencing by starting from the focal individual whose haplotypes are most frequent in the population. A potentially better strategy may be to select how many and which focal families to sequence across the whole population. This strategy could additionally account for shared ancestry across focal individuals, which would improve the phasing accuracy of haplotypes when using family-based sequence phasing algorithms [e.g. AlphaFamSeq (Battagin and Hickey, unpublished)]. We are currently developing methods to consider this.

Increasing the number of possible sequencing coverages to select from will increase the number of possible sequencing scenarios. In our analyses, with a choice of coverages of 0, 1, 2, 5, 10 and 20x, 281,728 sequencing scenarios and (281,728)^50^ possible combinations of sequencing scenarios could be evaluated across the 50 focal families. Estimating the phasing accuracies for each member of a focal family given a sequencing scenario is not a limitation if fast and efficient algorithms [e.g. AlphaFamSeq (Battagin and Hickey, unpublished)] are used, but evaluating all possible sequencing scenarios and combinations is computationally intensive and time consuming. Instead, the algorithm searches and samples from this vast space to find a solution. We are currently developing and testing other algorithms for sampling from large search spaces within reasonable time frames.

### Downstream analysis of sequence data

The main downstream applications of sequence data are to genotype known variants and/or identify novel variants, conduct GWAS analyses and fine-map causative mutations for traits of interest. To do this, many studies conduct high-coverage sequencing (i.e. 20 to 30x) on a few key individuals in the population (e.g. the key ancestors approach [4, 11–13]). Our results suggest that a better strategy to sequence and phase as many of the population haplotypes could be to sequence more individuals at a range of high, medium and low coverages. Many studies assume that low-coverage sequencing may not be sufficient for accurate variant identification, which would be true if a small number of individuals was sequenced. If a large number of individuals (e.g. a few hundred/thousand) was sequenced at low coverage, variant identification at the level of the population is possible, and has been demonstrated and successfully implemented in a number of studies (e.g. with 0.1x and 1x sequencing strategies [16, 26, 27]). This approach could detect low minor allele frequency variants, especially if a large number of individuals is sequenced [26].

### Summary of use cases and availability

A variety of data types with different features can be used. Populations can be unrelated, partially or highly related. Input data can be different densities and types (e.g. true or imputed genotypes, haplotypes or sequence). For example in practice, all individuals genotyped at high-density could be phased using AlphaPhase and all individuals genotyped at low-density could be imputed using AlphaImpute [16–19]. The options to provide a file of individuals that (a) have already been sequenced (thus their haplotypes are already accounted for), (b) should not be sequenced because they have no sample available (thus their haplotypes could be sequenced in other individuals), and (c) prior sequencing coverage (so that if an individual is selected to be sequenced at 10x but has already 2x sequence data, this is subtracted from the cost of sequencing this individual), is available. In addition, any non-linearity in sequencing costs that may arise due to different pricing structures for different sequencing technologies can be accounted for. Algorithm 2 is currently limited to populations with pedigree data and we are developing ways to extend this to populations without pedigree data. AlphaSeqOpt is available for download at http://www.alphagenes.roslin.ed.ac.uk/alphaseqopt/ along with a detailed user manual.

## Conclusions

We present two algorithms to select focal individuals whose haplotypes are more frequent in the population and to allocate a fixed sequencing budget across focal families to enable phasing of sequenced population haplotypes. The final aim of both algorithms is that as much as possible of the population haplotype diversity is sequenced and phased at the sequence level for a given fixed sequencing budget.

## Additional files

**Additional file 1: Figure S1.** Expected haplotype phasing accuracy against the sequencing investment on a focal family as estimated using AlphaFamSeq (Battagin and Hickey, unpublished). Description: AlphaFamSeq is a family-based method that performs phasing and imputation of markers using variable coverage sequence data. The algorithm requires sequence observations and at least three generations of pedigree as input. It first performs phasing using the sequence data of an individual itself, then refines and improves the phasing using sequence data from the individual’s parents and grandparents, builds a family level consensus haplotype and uses this to improve the phase and impute missing information for all individuals in a family of seven members.

**Additional file 2.** Detailed implementation of the algorithms.

**Additional file 3.** Choice of haplotype length.

**Additional file 4: Table S1.** Standardised cumulative proportion of times that haplotypes of the top 50 focal individuals selected by AlphaSeqOpt, the key ancestors approach (PEDIG) or the two haplotype-based approaches of Bickhart et al. [15] and Gusev et al. [16] appear in the rest of the population. **Table S2**. Standardised cumulative proportion of times that haplotypes of the top 200 focal individuals selected by AlphaSeqOpt, the key ancestors approach (PEDIG) or the two haplotype-based approaches of Bickhart et al. [15] and Gusev et al. [16] appear in the rest of the population. **Table S3**. The cumulative sum of the pedigree-inferred expected marginal contributions of the top 50 and 200 focal individuals selected by the key ancestors approach (implemented in the PEDIG software) for pedigrees of 5, 10, 15, 30 and 50 generations.

**Additional file 5: Figures S2, S3, S4, S5 and S6.** Percentage of the top 200 focal individuals selected by AlphaSeqOpt, PEDIG, or the two haplotype-based methods of Bickhart et al. [15] and Gusev et al. [16] against the number of generations for simulated pedigrees of 5 (Figure S2), 10 (Figure S3), 15 (Figure S4), 30 (Figure S5) and 50 (Figure S6) generations.
