# Supplementary figures and images for: A method for the allocation of sequencing resources in genotyped livestock populations
Source: Genet Sel Evol. 2017 May 18;49:47. doi: 10.1186/s12711-017-0322-5 (PMC5437657; doi:10.1186/s12711-017-0322-5)

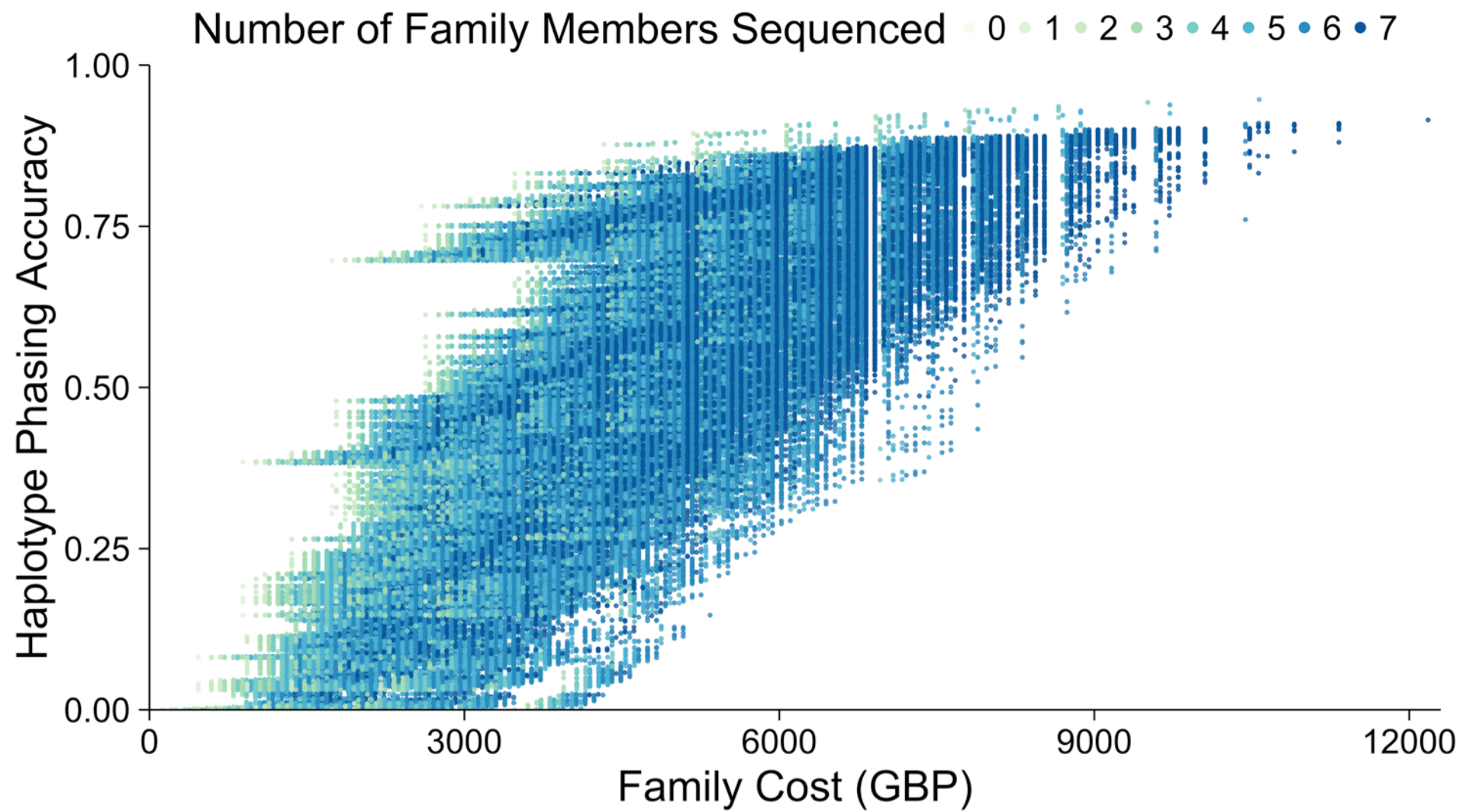

Supplement: Supplementary file 1 — Additional file 1: Figure S1. Expected haplotype phasing accuracy against the sequencing investment on a focal family as estimated using AlphaFamSeq (Battagin and Hickey, unpublished). Description: AlphaFamSeq is a family-based method that performs phasing and imputation of markers using variable coverage sequence data. The algorithm requires sequence observations and at least three generations of pedigree as input. It first performs phasing using the sequence data of an individual itself, then refines and improves the phasing using sequence data from the individual’s parents and grandparents, builds a family level consensus haplotype and uses this to improve the phase and impute missing information for all individuals in a family of seven members. [file 12711_2017_322_MOESM1_ESM.pdf]

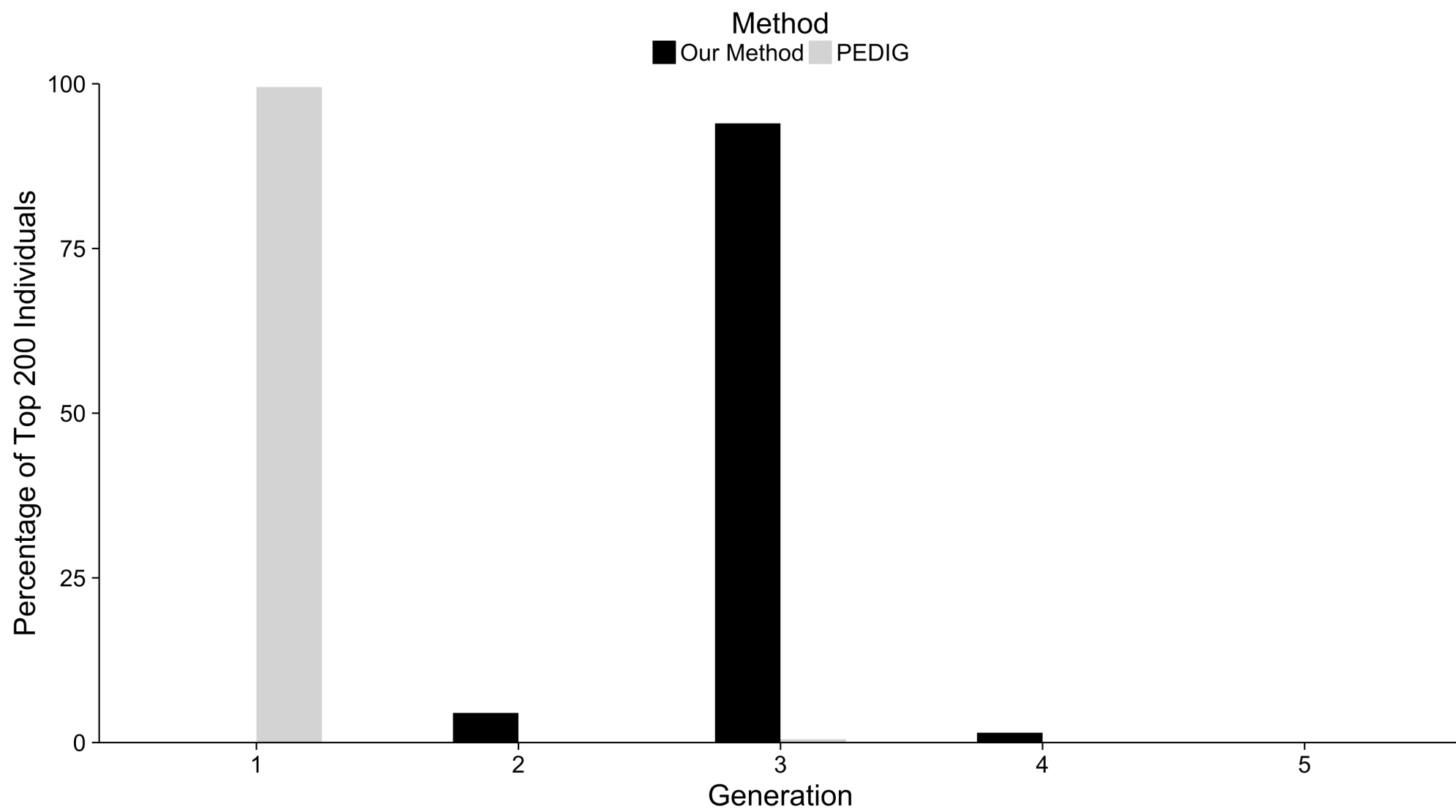

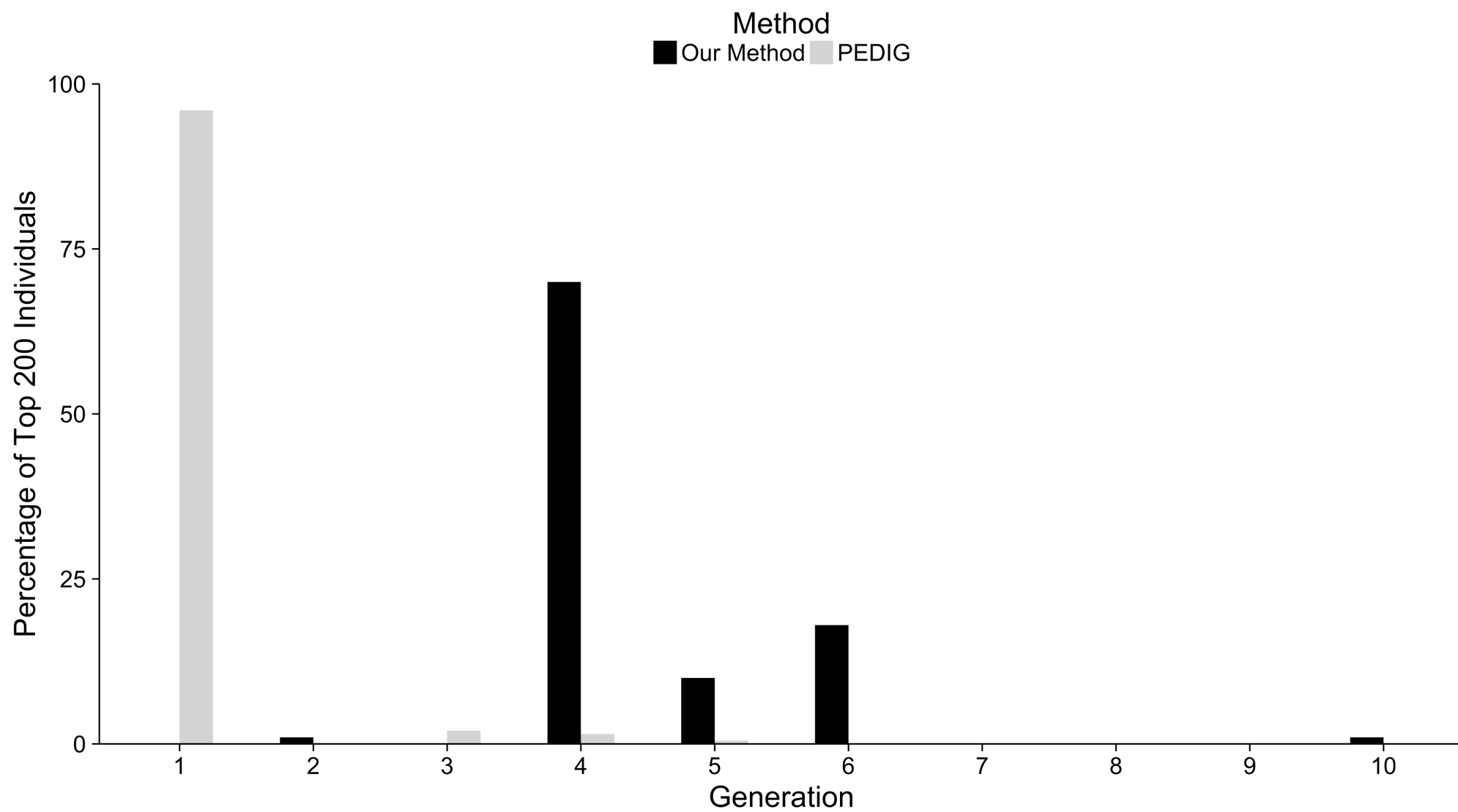

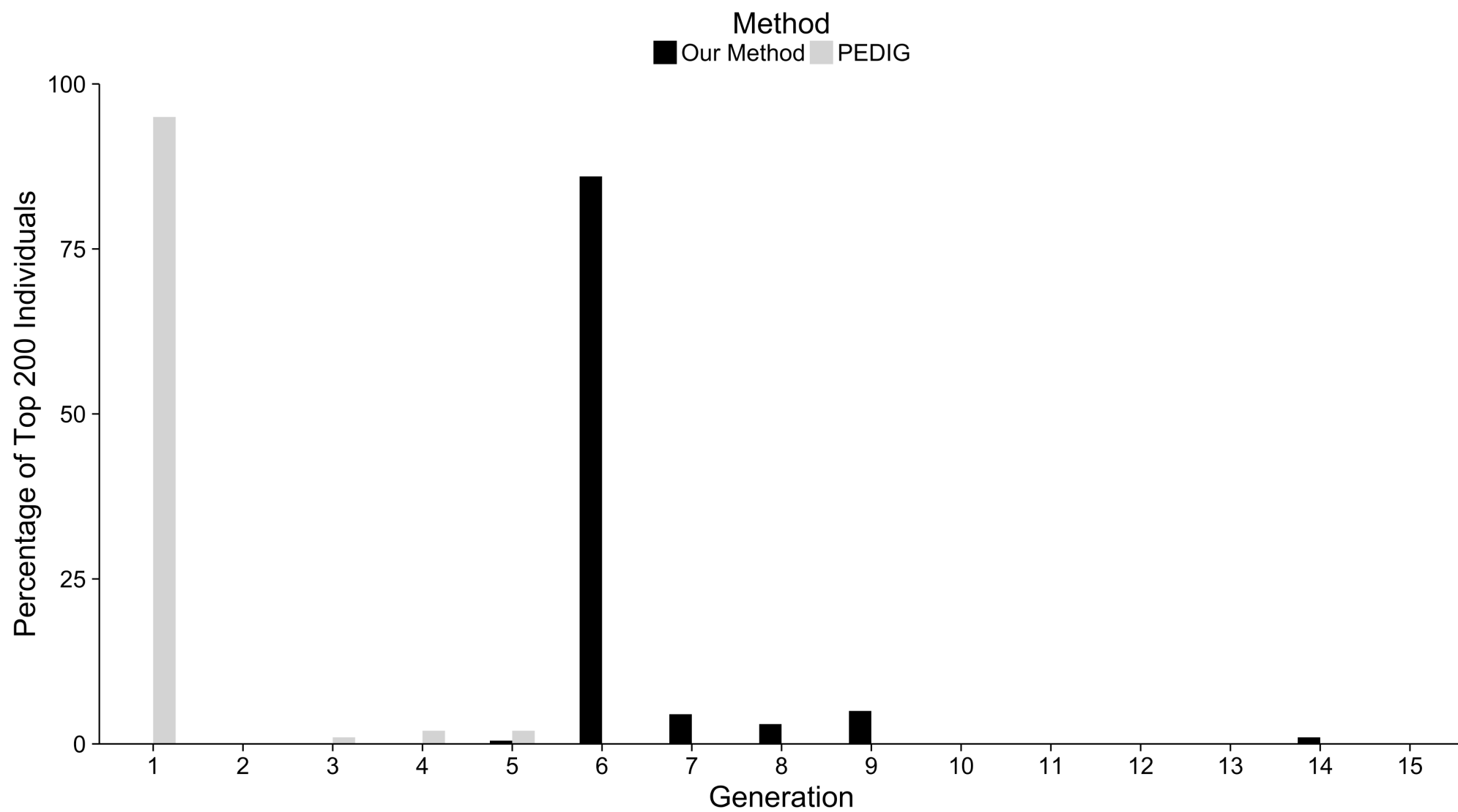

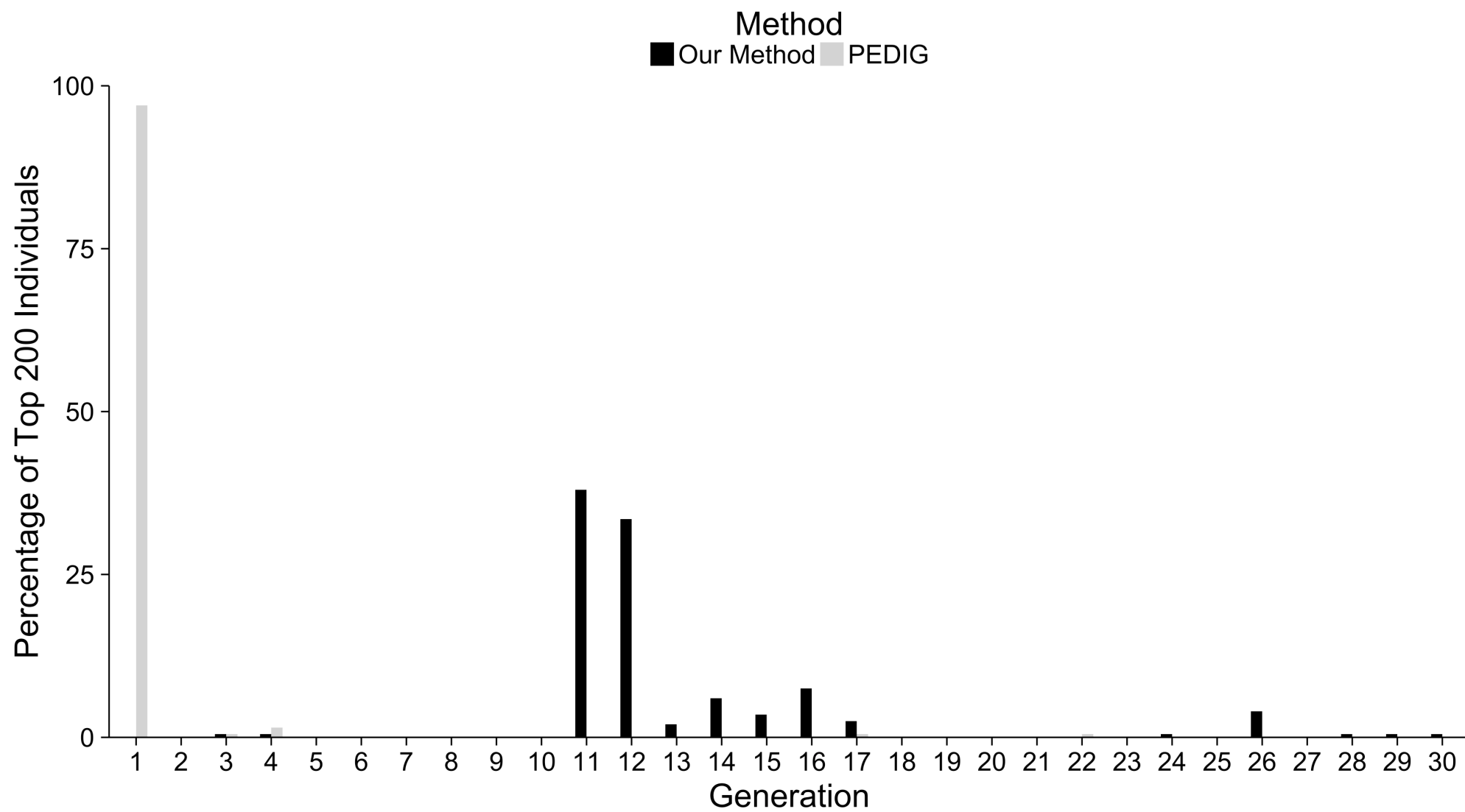

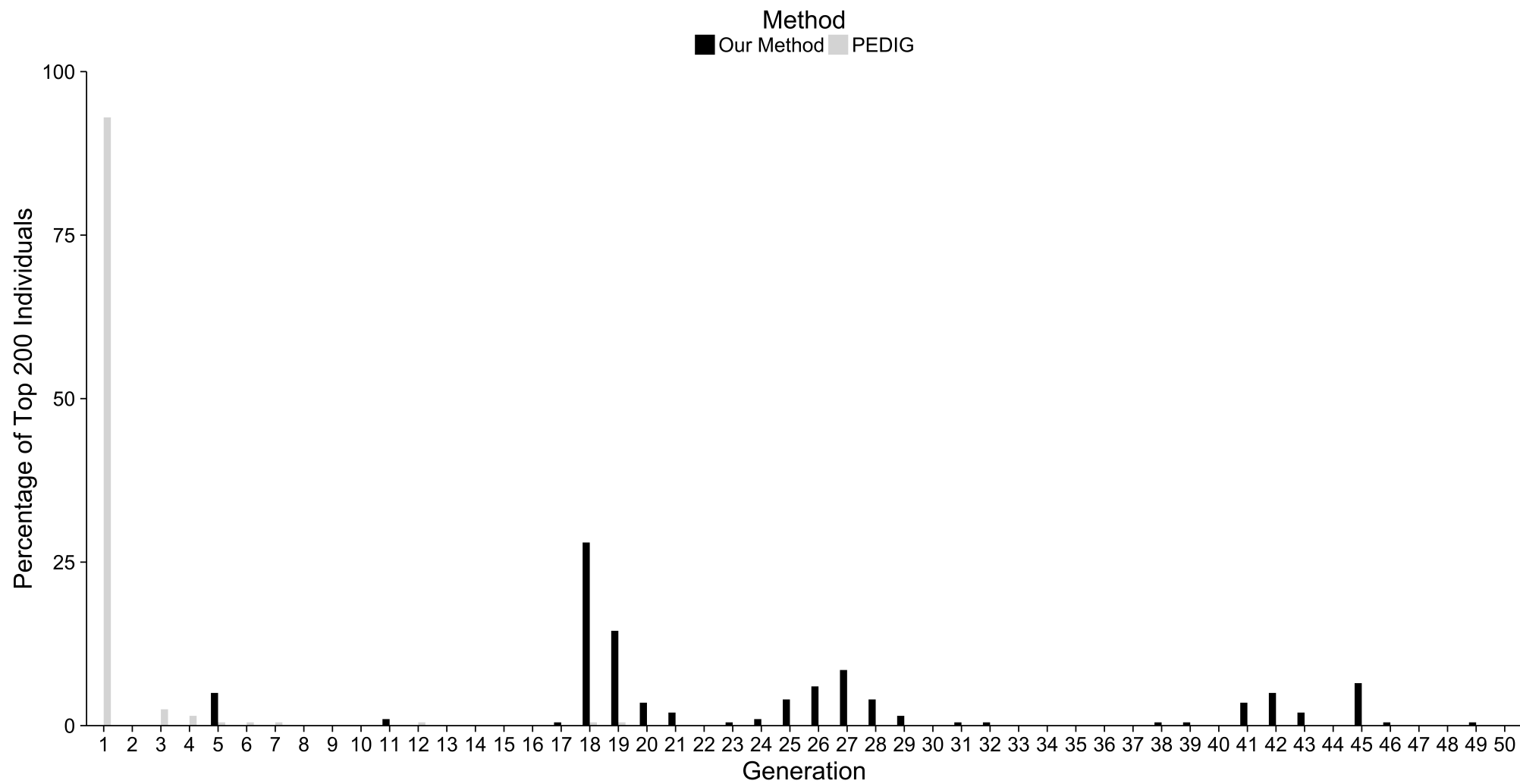

Supplement: Supplementary file 5 — Additional file 5: Figures S2, S3, S4, S5 and S6. Percentage of the top 200 focal individuals selected by AlphaSeqOpt, PEDIG, or the two haplotype-based methods of Bickhart et al. [15] and Gusev et al. [16] against the number of generations for simulated pedigrees of 5 (Figure S2), 10 (Figure S3), 15 (Figure S4), 30 (Figure S5) and 50 (Figure S6) generations. [file 12711_2017_322_MOESM5_ESM.pdf]
