# Supplementary material for: A method for the allocation of sequencing resources in genotyped livestock populations
Source: Genet Sel Evol. 2017 May 18;49:47. doi: 10.1186/s12711-017-0322-5 (PMC5437657; doi:10.1186/s12711-017-0322-5)
Supplement: Supplementary file 2 — Additional file 2. Detailed implementation of the algorithms. [file 12711_2017_322_MOESM2_ESM.docx]

# Supplementary File 1

## Input parameters, options and program features

The two algorithms for optimal distribution of sequencing resources in a population have been implemented in the software AlphaSeqOpt, which is part of the AlphaSuite package (<http://www.alphagenes.roslin.ed.ac.uk/>alphaseqopt/). A detailed user manual may also be found at this link.

The algorithms require the following inputs:

- A way of determining the haplotypes present and shared in the population. This can be in the form of:
  - Partially or fully phased genotypes
  - Partially or fully phased sequence data
  - Coded haplotype IDs carried by each individual
- Pedigree file of relationships across all individuals with genotypes
- File of possible sequencing scenarios
- File of phasing accuracies for each sequencing scenario
- Specification file

Optional files which could be given are:

- Individuals in the pedigree not to sequence
- Individuals with previous sequence information available
- Previous sequencing coverage and costs associated with prior sequencing data

Using the specification file, the user can provide / manipulate:

- Number of chromosomes to analyse
- If using phased genotypes to determine shared haplotypes:
  - Number of SNP genotypes per chromosome
  - Number of SNPs to define a haplotype (core length)
  - Thresholds for the inclusion of individuals and SNPs for determining the set of cores for each chromosome
  - Thresholds to compare and define shared haplotypes between individuals:
    - Minimum proportion of identity
    - Number of tolerated mismatches
- If using *apriori* determined haplotype IDs to determine shared haplotypes:
  - Number of cores in each chromosome
- Number of focal families to select for sequencing
- Total sequencing budget
- Vector of coverages to sample sequence coverages for the focal families
- Number of optimisation rounds to run to sample coverages and identify the optimum scenario to sequence the focal families
- Cost of library preparation per individual
- Cost of sequencing one individual at 1x

## Detailed implementation of the algorithms

AlphaSeqOpt is written in Fortran with object oriented coding to maximise performance and speed with large datasets.

**The steps in Algorithm 1 are expanded on below:**

Note: If phased genotype/sequence data are supplied then the algorithm looks for a file for each chromosome within a directory called “Phase” and starts from step 1. If haplotype IDs are supplied then the algorithm looks for a file for each chromosome within a directory called “Cores” and starts from step 5. The type of genomic information supplied is specified in the specifications file.

1. To determine the set of individuals and SNPs to use as the starting point for the construction of the haplotype library the input data is first filtered based on the thresholds set by the user.
   1. Individuals that pass the user’s choice of threshold for proportion of phased data are used as the base group of individuals to determine the set of haplotypes that are segregating in each core.
   2. SNPs are filtered so that only SNPs that pass the user’s choice of threshold for proportion of phased data are used to determine the haplotypes segregating in a core.
2. Using this filtered dataset, each chromosome is split into a set $m$ “cores” (i.e. haplotypes). The length of each core is constant and defined by the user in the specifications file as the number of SNPs per core. If the number of SNPs exceeding the threshold for phasing does not equally divide by the length of each core, core lengths are adjusted and excess SNPs are added to the ends of each core. For example, with 1010 SNPs and a core length of 100, the first 10 cores would have 101 SNPs and the remaining 90 would have 100 SNPs.
3. Individuals that pass the threshold for phasing are iterated over and their haplotypes within each core are assigned an ID and added to the haplotype library. If the haplotype has already been seen, the haplotype ID assigned to that individual is the pre-set haplotype ID.
4. Individuals that did not pass the threshold for phasing are iterated over. If the haplotypes that they carry are already present in the haplotype library then they are assigned that haplotype ID. If their haplotypes are not present in the library but the haplotype that they carry is well-phased (i.e. exceeds the haplotype phasing threshold specified by the user) then this haplotype is added to the library. If the haplotype of the individual cannot be determined due to an excess of missing information, this individual’s haplotype in that core is assigned missing (-99).
5. All individuals are iterated over. For each chromosome, core and haplotype in the haplotype library, the frequency of that haplotype in the population is calculated. Haplotypes are ordered based on frequency.
6. All individuals are iterated over and the number of times a given individual shares its haplotypes with other individuals in the population is determined.
7. Individuals are sorted according to this count and the top individual is selected as a focal individual for sequencing.
8. The haplotypes carried by this focal individual is masked in all other individuals under the assumption that sequencing and phasing its haplotypes would enable the imputation of these haplotypes into individuals that share these haplotypes.
9. Steps 5-8 are repeated to generate a list of the $k$ focal individuals for sequencing. Since $k$ is user-defined, this process can be repeated until all haplotypes in the population would be sequenced.

**The steps in Algorithm 2 are expanded on below:**

1. The proportion of haplotypes carried by the top $p$ focal individuals and their six immediate ancestors are taken in as input. A population “sequencing pool” is constructed and individuals that are shared between families are determined to prevent redundancy and errors in later steps of the algorithm.
2. For each individual in the sequencing pool, a sequencing coverage from a user-defined list of possible coverages is sampled. Sampling is performed based on the multinomial probabilities of sequencing an individual at a defined coverage. The probabilities are obtained by logit transforms of the internal problem representation in the differential evolution algorithm.
3. For each focal individual, the sampled coverage for itself and its six immediate ancestors is determined. This is defined as the “family sequencing scenario”. Family sequencing scenarios are also supplied by the user via a file whose path is specified in the specifications file. This file should contain all possible family sequencing scenarios given the possible coverages and should have 7 columns containing possible sequencing coverages for each family member.
4. Based on the family sequencing scenario, the following is calculated:
   1. The proportion of population haplotypes that would be accurately phased. This is calculated as the product of the proportion of population haplotypes carried by each member and the phasing accuracy for each member given the sampled sequencing coverage (AlphaFamSeq, Battagin and Hickey, in preparation). The phasing accuracies for each family sequencing scenario are supplied by the user via a file whose path is specified in the specifications file. This file should contain all possible accuracies for each family sequencing and should have 7 columns containing phasing accuracies for each family member.
   2. The cost of that family sequencing scenario. The cost of sequencing each member of a family takes into account pre-existing DNA libraries and/or sequence data for some individuals. Information on existing sequence data can be supplied in a file whose path should be provided in the specifications file.
5. Sum the costs of the selected set of family sequencing scenarios across all focal families. The overall cost takes into account that some individuals are part of multiple focal families and the cost of sequencing them should only be accounted for once.
6. Compute a “goodness criterion” for this combination of family sequencing scenarios. The criterion takes into account:
7. The proportion of population haplotypes that would be phased at the sequence level (as defined by algorithm 1).
8. The accuracy of phasing the haplotypes of focal individual given sampled sequencing scenarios (AlphaFamSeq, Battagin and Hickey, in preparation).
9. The fixed sequencing budget. If the overall total cost is above the budget then this combination of sequencing scenarios is penalised.

The criterion is calculated as the sum of the product of the proportion of population haplotypes carried by each family member and the accuracy of phasing the haplotypes of each family member, all divided by the maximum proportion of population haplotypes captured by all individuals in the sequencing pool.

1. Steps 2-5 are repeated $m$ times, where $m$ is the number of optimisation rounds defined by the user. The option to stop the algorithm after $n$ rounds of no change is also provided in the specifications file.

## Advantages of the algorithms

The algorithms have been implemented for fast use in large populations and datasets. The advantages of the implementation of the algorithms are:

- The software can utilise data in different formats, including:
  - Sequence data
  - SNP genotypes
  - Pre-determined haplotypes
  - Imputed / non-imputed data
- Coded for fast usage by:
  - *Apriori* pruning uninformative data points using user-defined thresholds for population haplotype library construction
  - Efficient storage of imputed genotype/sequence data by storing haplotype identifiers rather than phased genotypes
  - Coded for use on parallelised clusters
