# Supplementary material for: A method for the allocation of sequencing resources in genotyped livestock populations
Source: Genet Sel Evol. 2017 May 18;49:47. doi: 10.1186/s12711-017-0322-5 (PMC5437657; doi:10.1186/s12711-017-0322-5)

**Appendix 1**

**Optimising the choice of haplotype lengths**

The choice of which individuals should be sequenced depends on two factors: (i) the ability to identify the true haplotypes of an individual; and (ii) the ability to correctly infer haplotype sharing. The ability to identify the haplotypes an individual carries and shares is influenced by two factors: (i) the density of genomic information; and (ii) the number of SNPs used to define a core (i.e. core length).

The effect of these two parameters on the ability to correctly infer haplotypes is shown in Figure A1.1. Figure A1.1 shows that the sampling of SNPs across the genome on the SNP chip and the core length set within the program may infer that two individuals carry the same haplotype in a region of the genome, despite having different haplotypes at the full genome level.

To determine the optimal core length to be used with different SNP chip densities, additional simulations were run to obtain sequence, pedigree, QTN and phenotypes for fifteen generations, using the Markovian Coalescent Simulator [19] and AlphaSim [20,21].

A single chromosome of approximately 25,000 segregating sites and 100 cM in length was simulated. Segregating positions along the chromosome were randomly selected as SNPs to form six SNP chips of densities 1,500, 2,000, 3,000, 5,500, 15,000 and 20,000 SNPs. The chromosome was split into cores of different sizes depending on the number of SNPs within defined lengths of 0.5, 1, 2.5, 5, 10, 15, 20, 25, 50 and 100 cM. For example, with a chip density of 20,000 SNPs, core lengths ranged from all SNPs in a single 100 cM core to 90 SNPs in 0.5cM cores.

For each SNP chip and core length, 1,000 individuals were sampled across the pedigree. For each individual, the haplotypes it carries and shares with other individuals in the population as defined by the SNP chip and core length was determined. For each individual, the full genome was then split into the same number of cores as defined by the SNP chip density, and the true haplotypes carried and shared by individuals were determined. The proportion of times that the sharing of haplotypes between two individuals was correctly inferred by the SNP chip was determined. This process was repeated 10 times to obtain an average value.

Increasing the core length increases the proportion of haplotypes correctly identified and shared across individuals. This was true across all SNP chip densities. This is shown in Figure A1.2, which is a plot of the percentage of correctly inferred shared haplotypes against the core length in cM for the six SNP chips of different densities. On average across all SNP chip densities, 1.02 times more of the haplotypes were correctly inferred as shared when the core length was set to 100 cM compared to 50 cM (99.52 vs. 98.03), 1.07 times more were correctly inferred when the core length was set to 100 cM compared to 25 cM (99.52 vs. 93.38), 1.39 times more were correctly inferred when the core length was set to 100 cM compared to 10 cM (99.52 vs. 71.60) and 1.77 times more were correctly inferred when the core length was set to 100 cM compared to 0.5 cM (99.52 vs. 56.31).

Figure A1.2 also shows that a core length of 25 cM (i.e. one quarter of the chromosome) was required to ensure that at least 90% of the haplotypes were correctly inferred as shared across individuals, which is what we used in our analyses.

Figure A1.1 – The effect of (a) SNP sampling and (b) core length on the ability to correctly infer shared haplotypes between two individuals.


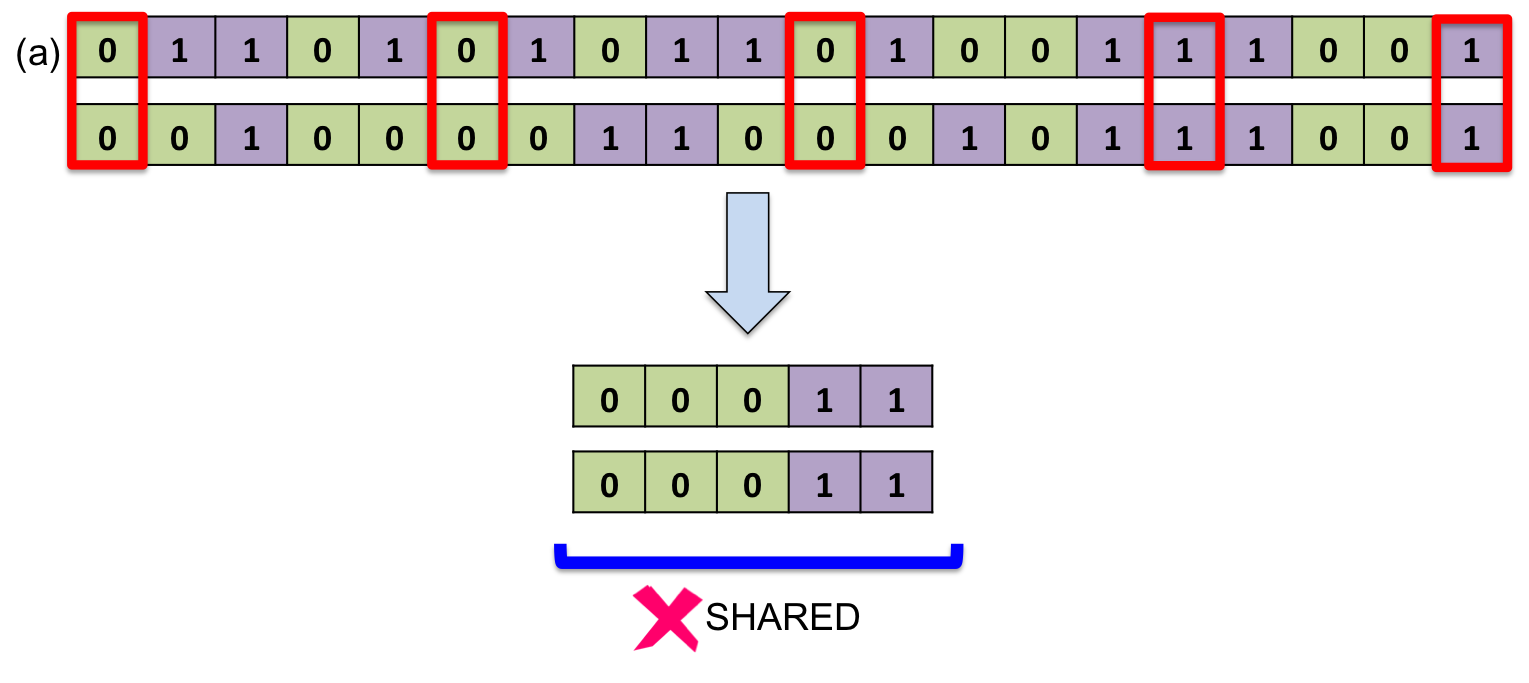

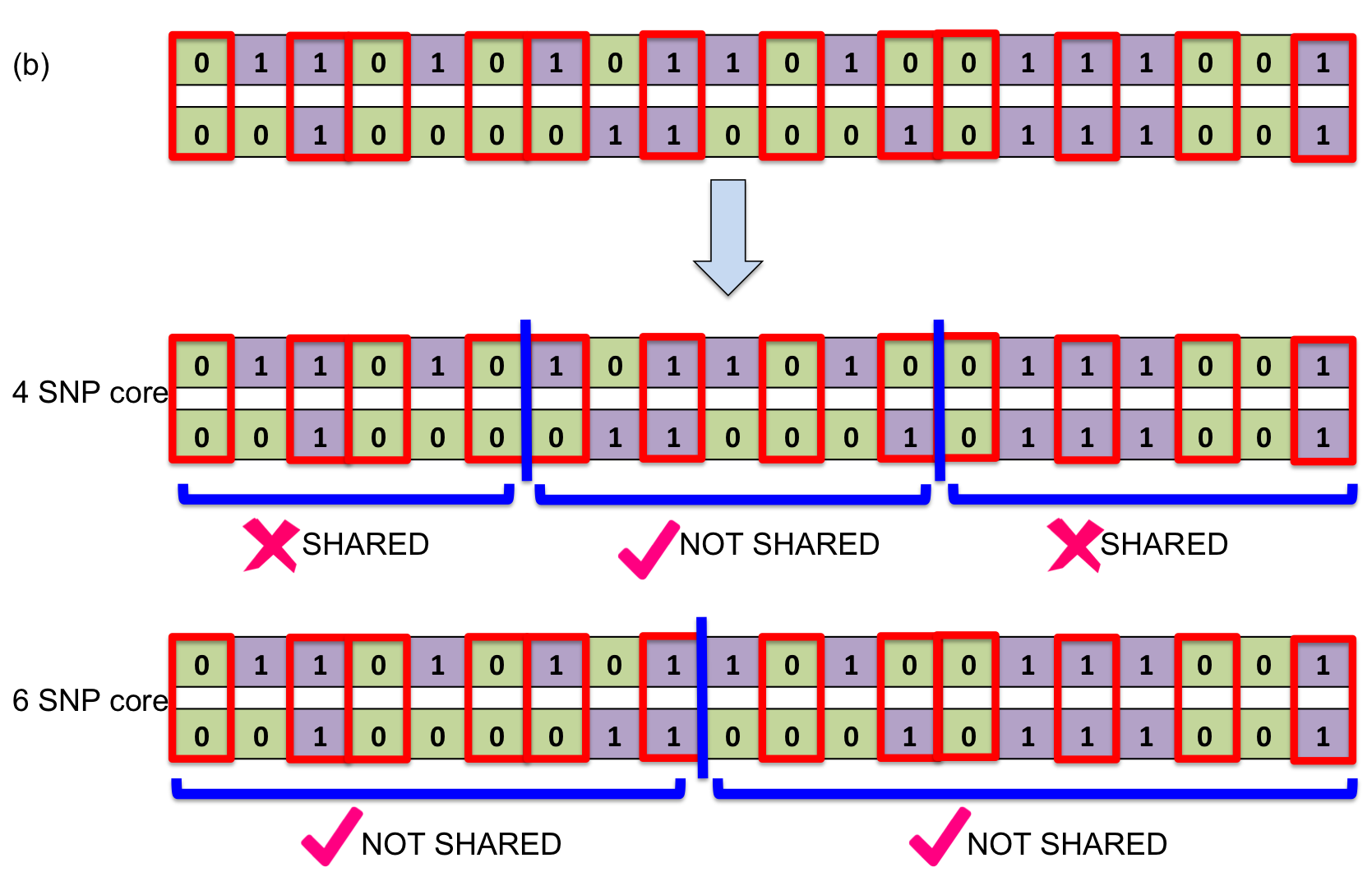


Figure A1.2 – The proportion of haplotypes correctly identified and shared across individuals with core lengths ranging from 0.5 cM to 100 cM and SNP chips of low, medium and high density.


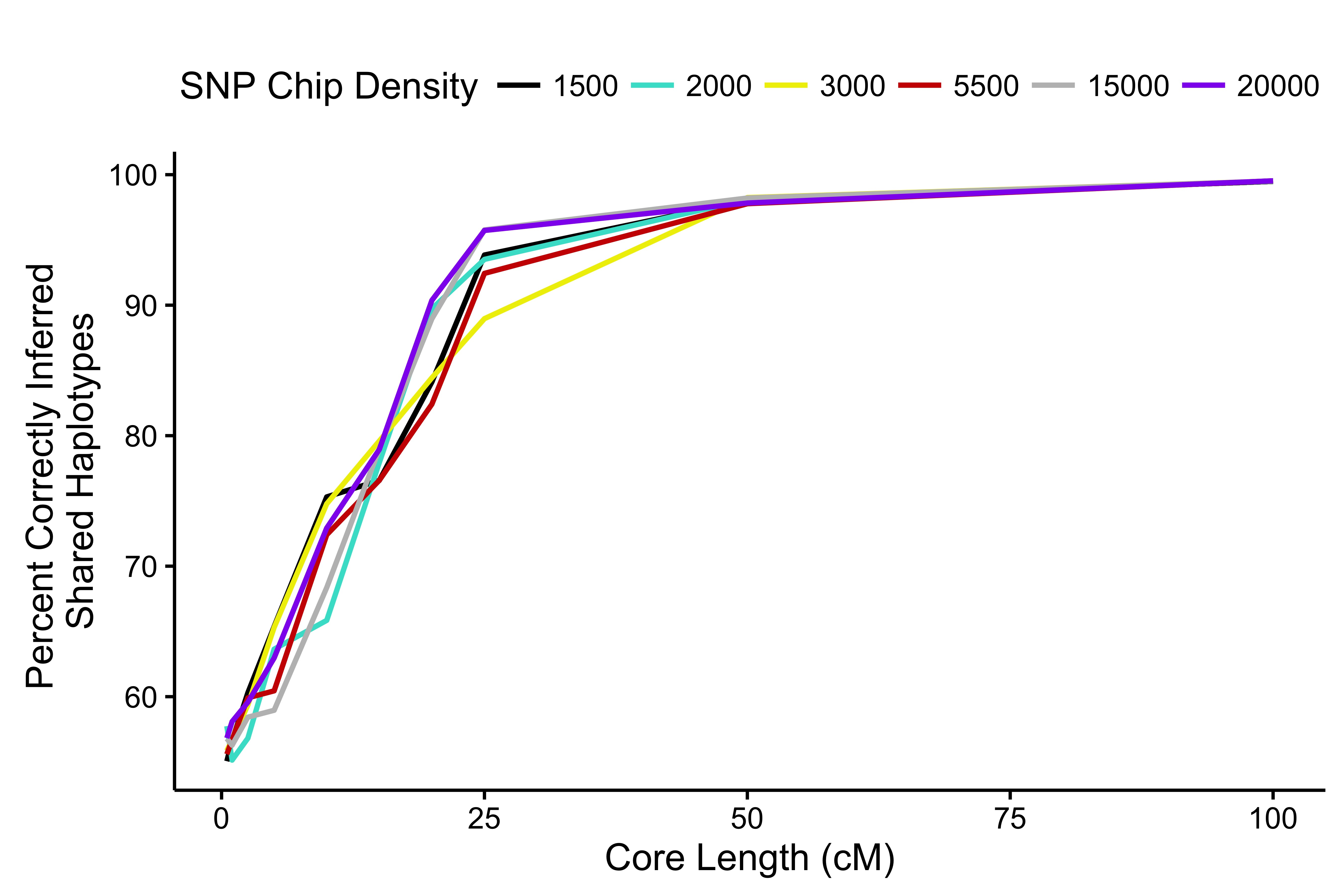

Supplement: Supplementary file 3 — Additional file 3. Choice of haplotype length. [file 12711_2017_322_MOESM3_ESM.docx]
