# Supplementary material for: A method for the allocation of sequencing resources in genotyped livestock populations
Source: Genet Sel Evol. 2017 May 18;49:47. doi: 10.1186/s12711-017-0322-5 (PMC5437657; doi:10.1186/s12711-017-0322-5)
Supplement: Supplementary file 4 — Additional file 4: Table S1. Standardised cumulative proportion of times that haplotypes of the top 50 focal individuals selected by AlphaSeqOpt, the key ancestors approach (PEDIG) or the two haplotype-based approaches of Bickhart et al. [15] and Gusev et al. [16] appear in the rest of the population. Table S2. Standardised cumulative proportion of times that haplotypes of the top 200 focal individuals selected by AlphaSeqOpt, the key ancestors approach (PEDIG) or the two haplotype-based approaches of Bickhart et al. [15] and Gusev et al. [16] appear in the rest of the population. Table S3. The cumulative sum of the pedigree-inferred expected marginal contributions of the top 50 and 200 focal individuals selected by the key ancestors approach (implemented in the PEDIG software) for pedigrees of 5, 10, 15, 30 and 50 generations. [file 12711_2017_322_MOESM4_ESM.docx]

**Supplementary File 3**

Supplementary Tables S1, S2 and S3

Supplementary Table S1 – Standardised cumulative proportion of times that haplotypes of the top 50 focal individuals selected by AlphaSeqOpt, the key ancestors approach (PEDIG) or the two haplotype-based approaches of Bickhart et. al. [15] and Gusev et. al. [16] appear in the rest of the population.

| **Pedigree size (Generations)** | **Our method** | **Key ancestors approach** | **Bickhart** | **Gusev** |
| --- | --- | --- | --- | --- |
| 5 | 7.09  (7.08-7.10) | 7.04  (7.03-7.05) | 5.55  (4.79-6.01) | 6.78  (5.97-7.44) |
| 10 | 6.31  (6.30-6.35) | 5.08  (5.07-5.10) | 5.03  (3.97-5.93) | 5.81  (4.95-6.62) |
| 15 | 5.92  (5.90-5.94) | 4.00  (3.98-4.01) | 5.06  (3.75-6.67) | 5.65  (4.45-7.11) |
| 30 | 5.01  (4.97-5.06) | 2.43  (2.42-2.44) | 4.22  (2.45-9.45) | 5.00  (3.43-10.37) |
| 50 | 4.85  (4.81-4.88) | 1.71  (1.71-1.72) | 4.50  (2.50-8.04) | 5.16  (3.64-8.37) |

Supplementary Table S2 – Standardised cumulative proportion of times that haplotypes of the top 200 focal individuals selected by AlphaSeqOpt, the key ancestors approach (PEDIG) or the two haplotype-based approaches of Bickhart et. al. [15] and Gusev et. al. [16] appear in the rest of the population.

| **Pedigree size (Generations)** | **Our method** | **Key ancestors approach** | **Bickhart** | **Gusev** |
| --- | --- | --- | --- | --- |
| 5 | 25.40  (25.35-25.45) | 20.21  (20.19-20.25) | 21.52  (19.26-23.66) | 20.85  (18.07-23.77) |
| 10 | 22.56  (22.47-22.65) | 14.09  (14.06-14.12) | 18.16  (13.98-22.83) | 17.94  (14.94-21.67) |
| 15 | 20.56  (20.50-20.62) | 10.92  (10.88-10.96) | 17.80  (14.36-21.30) | 17.18  (14.48-18.79) |
| 30 | 19.47  (19.31-19.63) | 6.50  (6.48-6.52) | 15.41  (9.71-35.86) | 14.75  (10.15-31.86) |
| 50 | 18.24  (18.12-18.36) | 4.07  (4.05-4.09) | 16.57  (11.11-30.34) | 16.30  (10.31-31.40) |

Supplementary Table S3 – The cumulative sum of the pedigree-inferred expected marginal contributions of the top 50 and 200 focal individuals selected by the key ancestors approach (implemented in the PEDIG software) for pedigrees of 5, 10, 15, 30 and 50 generations.

| **Pedigree size (Generations)** | **Top 50** | **Top 200** |
| --- | --- | --- |
| 5 | 0.473  (0.471-0.474) | 0.590  (0.588-0.590) |
| 10 | 0.268  (0.265-0.268) | 0.342  (0.338-0.342) |
| 15 | 0.181  (0.178-0.181) | 0.231  (0.227-0.231) |
| 30 | 0.092  (0.090-0.092) | 0.118  (0.116-0.118) |
| 50 | 0.056  (0.055-0.056) | 0.072  (0.071-0.072) |
